# Pyrrocidine, a molecular off switch for fumonisin biosynthesis

**DOI:** 10.1371/journal.ppat.1008595

**Published:** 2020-07-06

**Authors:** Minglu Gao, Anthony E. Glenn, Xi Gu, Trevor R. Mitchell, Timothy Satterlee, Mary V. Duke, Brian E. Scheffler, Scott E. Gold

**Affiliations:** 1 Department of Plant Pathology, University of Georgia, Athens, Georgia, United States of America; 2 USDA, ARS, US National Poultry Research Center, Toxicology & Mycotoxin Research Unit, Athens, Georgia, United States of America; 3 Institute of Bioinformatics, University of Georgia, Athens, Georgia, United States of America; 4 USDA, ARS, Genomics and Bioinformatics Research Unit, Stoneville, Mississippi, Untied States of America; Purdue University, UNITED STATES

## Abstract

*Sarocladium zeae* is a fungal endophyte of maize and can be found co-inhabiting a single seed with *Fusarium verticillioides*, a major mycotoxigenic food safety threat. *S*. *zeae* produces pyrrocidines A and B that inhibit the growth of *F*. *verticillioides* and may limit its spread within the seed to locations lacking *S*. *zeae*. Although coinhabiting single seeds, the fungi are generally segregated in separate tissues. To understand *F*. *verticillioides’* protective physiological response to pyrrocidines we sequenced the *F*. *verticillioides* transcriptome upon exposure to purified pyrrocidine A or B at sub-inhibitory concentrations. Through this work we identified a *F*. *verticillioides* locus *FvABC3* (FVEG_11089) encoding a transporter critical for resistance to pyrrocidine. We also identified *FvZBD1* (FVEG_00314), a gene directly adjacent to the fumonisin biosynthetic gene cluster that was induced several thousand-fold in response to pyrrocidines. *FvZBD1* is postulated to act as a genetic repressor of fumonisin production since deletion of the gene resulted in orders of magnitude increase in fumonisin. Further, pyrrocidine acts, likely through FvZBD1, to shut off fumonisin biosynthesis. This suggests that *S*. *zeae* is able to hack the secondary metabolic program of a competitor fungus, perhaps as preemptive self-protection, in this case impacting a mycotoxin of central concern for food safety.

## Author summary

The fungal food safety threat, *Fusarium verticillioides*—producer of the deadly fumonisin mycotoxins, is a seed borne pathogen of corn. Another fungus, *Sarocladium zeae*, also lives in corn seed. *S*. *zeae* produces compounds called pyrrocidines that inhibit the growth of *F*. *verticillioides*. Here we describe the *F*. *verticillioides* transcriptional response upon exposure to pyrrocidines. By making deletion mutants in highly upregulated genes we determined the gene crucial for *F*. *verticillioides* resistance to pyrrocidine. Another gene, which was the most highly upregulated, suppresses fumonisin production. Additionally, we found that pyrrocidine effectively eliminates fumonisin production in wild *F*. *verticillioides*. This provides potential strategies for development of biological and/or chemical control to eliminate fumonisin contamination of food and feed.

## Introduction

As one of the most notorious mycotoxigenic plant pathogens, *Fusarium verticillioides* poses a serious worldwide threat to the health of maize as well as livestock and humans. *F*. *verticillioides* can elicit severe kernel rot symptoms, which are often coupled with high levels of mycotoxin contamination [1]. Production of *F*. *verticillioides* mycotoxins, predominantly the fumonisins, are associated with animal toxicoses, such as leukoencephalomalacia in horses and pulmonary edema in swine [2,3]. Human health concerns associated with fumonisin exposure include potential birth defects, stunting and some cancers (Riley et al., 2019). Further, its endophytic life style increases difficulties for disease management, as the infected kernels often remain visually symptomless [1].

Along with *F*. *verticillioides* is another kernel endophyte, *Sarocladium zeae* (formerly known as *Acremonium zeae*) [4,5]. Early histopathological studies on “sound-appearing” maize kernels revealed that *F*. *verticillioides* primarily colonizes the pedicel and abscission layer of the developing maize seed, while *S*. *zeae* was often detected in the embryo and endosperm [6]. These observations describe an interesting phenomenon in which these two fungal endophytes may sympatrically co-inhabit the same seed but retain structural compartment or tissue specificity.

*S*. *zeae* produces the pyrrocidines, first described in 2002 for their antibacterial properties [7]. These lactam compounds, pyrrocidine A and B, also demonstrated *in vitro* inhibitory activity against *F*. *verticillioides*, as well as *Candida albicans* and *Aspergillus flavus*, which might impact the partitioning of *F*. *verticillioides* and *S*. *zeae* to different tissues within maize kernels [8,9]. Pyrrocidine A differs from pyrrocidine B by presence of a double bond in its lactam ring, resulting, by an unknown mechanism, in a higher toxicity of pyrrocidine A than B against a number of fungal and bacterial species [8,9]. The crucial role of this double bond in determining apoptosis inducing cytotoxicity against human acute promyelocytic leukemia HL60 cells [10]. Pyrrocidine A and B can both be detected in *S*. *zeae*-infested kernels through liquid chromatography-mass spectrometry (LC-MS), but the concentration of pyrrocidines during natural occurrence in maize seeds has not been described [8,9]. *S*. *zeae* is not reported to synthesize secondary metabolites harmful to plants, nor does it cause any ear or stem rot symptoms [11]. Collectively, these studies support a role of *S*. *zeae* as a “protective” endophyte and bring attention to its potential as a biological control agent [9].

The hypothesis that *S*. *zeae* employs pyrrocidines as metabolic weapons to exclude *F*. *verticillioides* from *S*. *zeae*-colonized seed structures is worthy of study, but how *F*. *verticillioides* genetically or biochemically responds to pyrrocidine exposure is unknown, as is whether such responses contribute to coexistence of the two endophytes in the plant. For example, since pyrrocidines contain lactam rings, do any of the *F*. *verticillioides* genes induced by the compounds encode lactamases? We recently reported a thorough inventory of fungal lactamases and their potential functions in the environment [12]. In order to define the possible defensive mechanisms by which *F*. *verticillioides* survives in the presence of pyrrocidines, we explored transcriptional responses via RNA sequencing in *F*. *verticillioides* upon exposure to purified pyrrocidine A or B at sub-inhibitory concentrations. Pyrrocidine A and B treatments shared 395 up-regulated and 130 down-regulated genes. Ten of the up-regulated genes were selected for functional characterization, and three of the genes proved to be particularly informative. Mutants in one of these genes, encoding a pleiotropic-drug resistance (PDR)-type ATP-binding cassette (ABC) transporter (FVEG_11089), showed dramatically increased sensitivity to pyrrocidine B. A co-upregulated adjacent gene encoding a putative transcription factor (FVEG_11090) was found to be dispensable for the induction of FVEG_11089. A gene encoding a putative zinc-binding dehydrogenase (FVEG_00314) was the most highly pyrrocidine-induced gene, and its deletion dramatically derepressed production of the fumonisins. Interestingly, FVEG_00314 is located directly adjacent to the *FUM21* gene encoding the *cis*-regulatory transcription factor for the fumonisin biosynthetic gene cluster. Finally, sub-fungitoxic levels of pyrrocidine acts as a powerful off switch for fumonisin biosynthesis.

## Results

### 1. Pyrrocidine A and B elicited differential gene responses in *Fusarium verticillioides*

**Fig 1:**
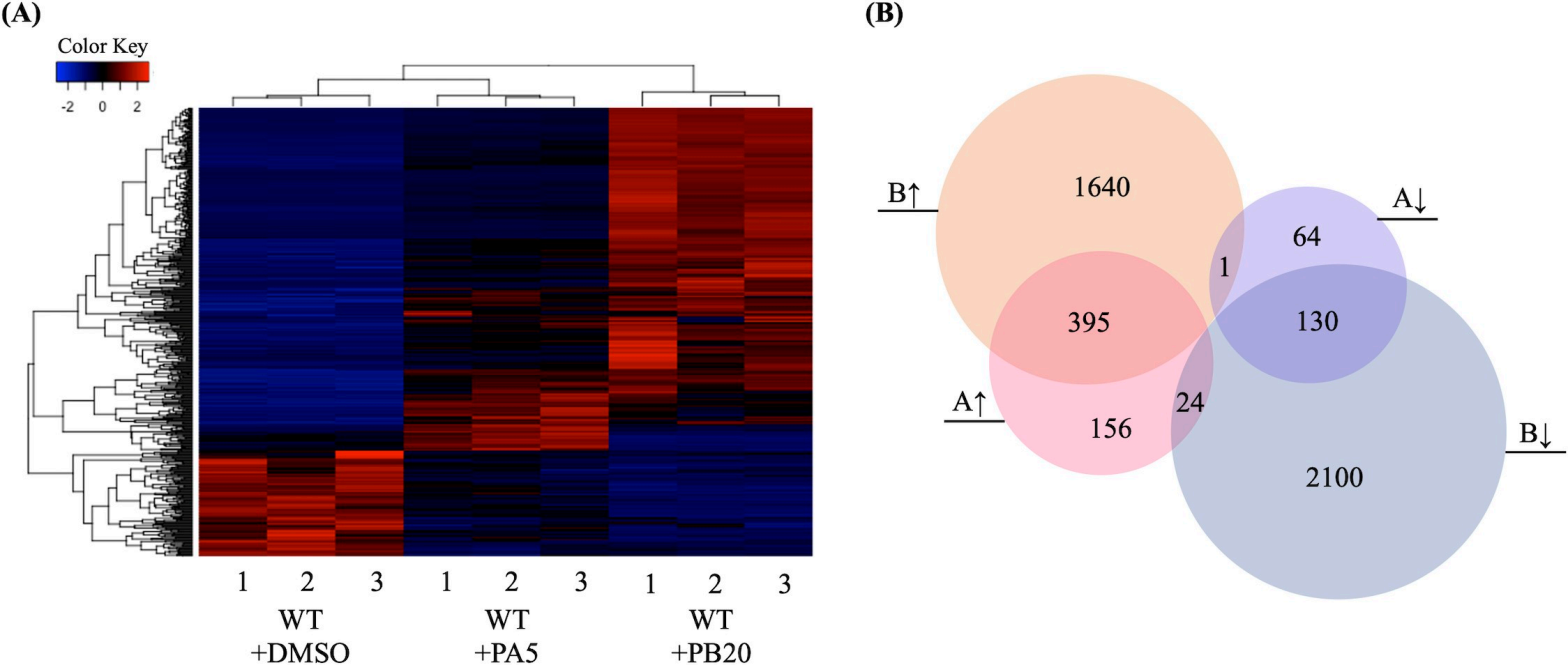
Pyrrocidine A and B elicit differential gene expression. (**A**) Heatmaps showing transcription levels of 4510 differentially expressed genes upon exposure to pyrrocidine A (PA, 5μg/mL) or pyrrocidine B (PB, 20μg/mL). The Y-axis represents genes that are clustered and colored by z-score. See colored key. The X-axis shows the 3 biological replicates of each treatment. (**B**) Venn diagram shows the number of genes with altered expression due to pyrrocidine A and/or B exposure. Each circle represents up- or down-regulation by pyrrocidine A or B, which is denoted by up/down arrows and A/B, respectively. Intersected regions represent genes regulated in both treatments, while the direction of regulation may vary.

To explore the transcriptional responses of *F*. *verticillioides* upon exposure to pyrrocidine A or pyrrocidine B, the transcriptome of wild-type *F*. *verticillioides* induced by either compound was sequenced and compared to that of a DMSO-treated control. The raw RNA-Seq reads of each biological replicate ranged from 14.2–21.9 million, of which 13.9–21.5 million reads were mapped to the reference genome of *F*. *verticillioides* (S1 Table) [13]. When applying two thresholds (first a false discovery rate-adjusted p-value < 0.05, and second a log2 fold change > 1 for up-regulated genes or < -1 for down-regulated genes), we identified 770 and 4290 differentially expressed genes upon exposure to pyrrocidine A and B, respectively, when compared to the DMSO only treated control. Similar levels of expression were observed among the biological replicates (Fig 1A). Pyrrocidine A and B treatments shared 395 up-regulated genes and 130 down-regulated genes. Only 25 genes were identified as up-regulated in one treatment and down-regulated in the other (Fig 1B). Considering the high similarity between the pyrrocidine A and B chemical structures, we focused on genes co-upregulated by both compounds for subsequent functional analyses.

### 2. Pyrrocidine-responsive genes were primarily enriched in metabolism, cellular transport, and cell rescue functions

**Fig 2:**
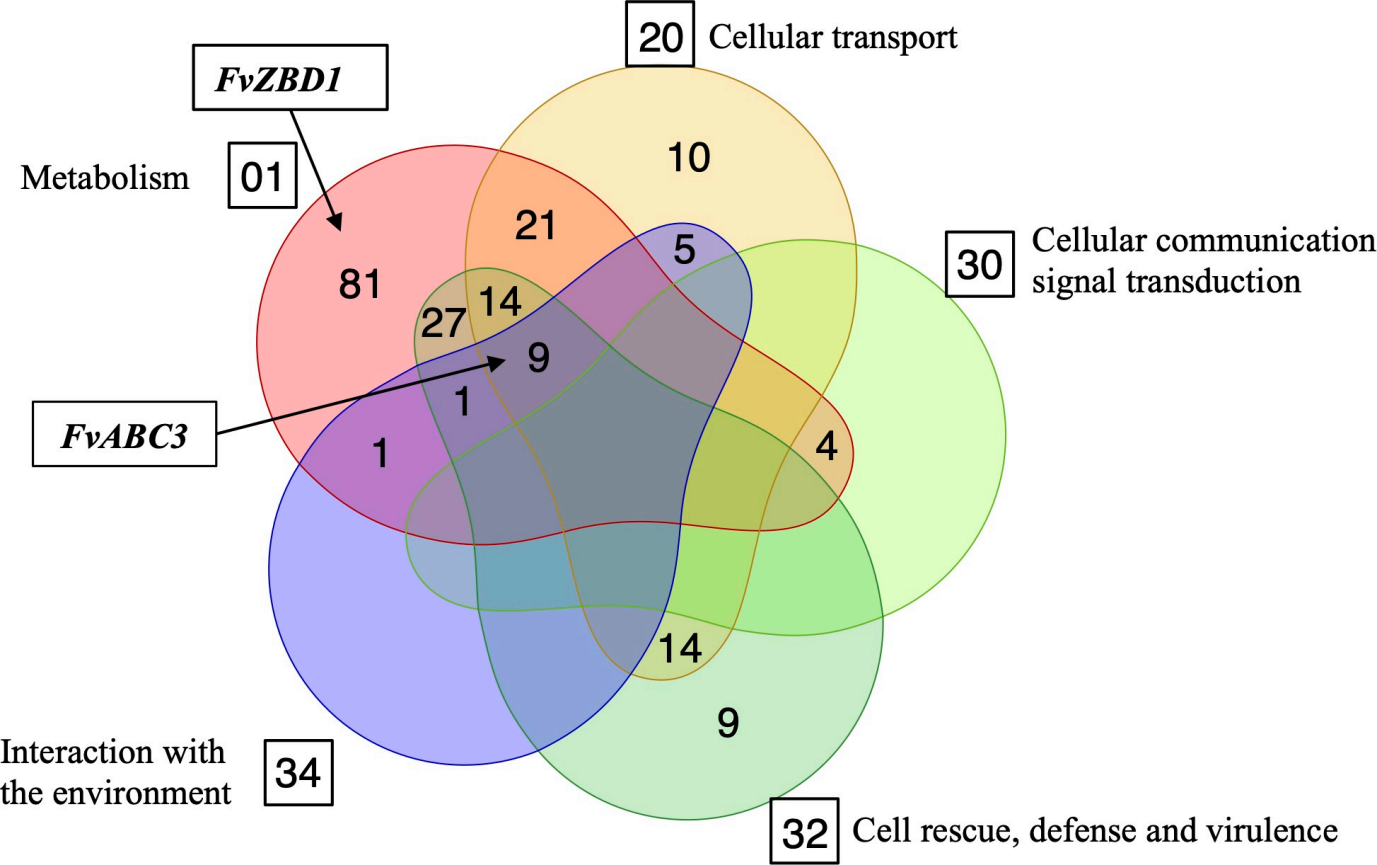
Functional catalog of pyrrocidine-responsive genes. Function enrichment analysis was conducted with MIPS FunCat Server (http://mips.helmholtz-muenchen.de/funcatDB/) [14]. The five club shapes correspond to the following MIPS functional categories: metabolism (01), cellular transport (20), cellular communication/signal transduction mechanism (30), cell rescue, defense and virulence (32), and interaction with the environment (34). The numbers of genes functionally enriched in the different categories are included, and genes with multifunctional enrichment are shown in overlapping regions. Of 525 pyrrocidine-responsive genes, 196 genes were functionally enriched in these five different categories after applying a filter of p-value < 0.05. *FvABC3* is one of nine genes that functionally span four different enriched categories (01, 20, 32, 34), and *FvZBD1* is functionally enriched only in metabolism along with another 80 genes.

To better understand the functional potential of genes responsive to both pyrrocidines A and B, the 395 up-regulated and 130 down-regulated genes were categorized using the MIPS Functional Catalogue [14], and enriched categories with p-value < 0.05 are shown in Fig 2. We identified 196 genes significantly enriched in the following functional categories with corresponding catalog numbers in parentheses: metabolism (01), cellular transport (20), cellular communication/signal transduction mechanism (30), cell rescue, defense and virulence (32), and interaction with the environment (34). The vast majority of the enriched genes (158/196) were associated with metabolism and cellular transport, while only nine genes were exclusively enriched in cell rescue, defense, and virulence (Fig 2). There were also nine genes showing significant enrichment in the four functional categories of (01), (20), (32), and (34). Among these was an ABC transporter-encoding gene (*FvABC3*, FVEG_11089) highly induced by both pyrrocidine A and B treatments (Table 1). Directly upstream of *FvABC3* is the co-induced *FvZEAR* (FVEG_11090), which encodes a putative transcription factor.

**Table 1:** Genes targeted for functional characterization.

Gene#	Annotation	Pyrrocidine A Exposure		Pyrrocidine B Exposure	
		p-value^a^	Log2 FC^b^	p-value	Log2 FC
FVEG_00314 (***FvZBD1***)	Alcohol dehydrogenase	1.57E-224	12.02	8.48E-228	12.08
FVEG_01675	Dioxygenase	7.18E-210	7.86	1.85E-217	7.98
FVEG_07235	NADH(P)-binding	2.49E-78	9.60	1.47E-63	8.57
FVEG_09038	Cytochrome P450	3.31E-48	7.48	9.67E-103	10.86
FVEG_11089 (***FvABC3***)	ABC transporter	0	5.63	0	8.88
FVEG_11090 (***FvZEAR***)	Zn(2)-Cys(6) transcription factor	9.02E-158	5.29	0	7.44
FVEG_13271	Short chain dehydrogenase	1.34E-111	10.36	3.52E-124	10.88
FVEG_13322	Short chain dehydrogenase	0.00313	1.52	2.68E-155	11.49
FVEG_17422	ABC transporter	1.90E-18	1.18	0	6.23
FVEG_17625	C2H2-type zinc finger	3.54E-80	2.93	0	8.30

^**a**^ p-value, false discovery rate-adjusted p-value

^**b**^ FC, fold change

### 3. Deletion of ten pyrrocidine-induced genes

To explore their relevance to pyrrocidine resistance, 10 *F*. *verticillioides* genes of interest from different FunCat categories were targeted for functional characterization. These 10 genes demonstrated strong and statistically significant induction by pyrrocidines (log2 fold change > 1 in pyrrocidine A treatment and > 6 in pyrrocidine B treatment, plus a false discovery rate-adjusted p-value < 0.01), low or nominal FPKM (Fragments Per Kilobase of transcript per Million mapped reads) values (< 100) in the control treatment, and high expression levels in the pyrrocidine B treatment (FPKM > 950) (Table 1). All ten of these genes targeted for deletion were confirmed by qRT-PCR to be differentially expressed in similar patterns to the RNAseq data (S3 Table).

*F*. *verticillioides* deletion mutants were generated with the OSCAR protocol [15,16] for each of the 10 target genes and validated by specific PCR assays for each gene and deletion construct and by quantitative PCR (qPCR) to determine copy number for the hygromycin resistance cassette to ensure the mutants had only single insertions (S1 and S2 Figs). Subsequent growth curve analyses were carried out by challenging the collection of mutants with pyrrocidine B at 20 μg/mL, the same concentration employed in RNA-Seq experiments. Initial screening of the deletion mutants in liquid PDB revealed that Δ*FvABC3* mutants exhibited almost no growth when exposed to pyrrocidine B, and that Δ*FvZBD1* mutants showed slightly reduced growth, while gene deletion strains for the other eight genes demonstrated no significantly enhanced sensitivity compared to the wild-type M-3125.

### 4. *FvABC3* significantly impacted the resistance to pyrrocidine B, whereas the adjacent transcription factor *FvZEAR* did not

**Fig 3:**
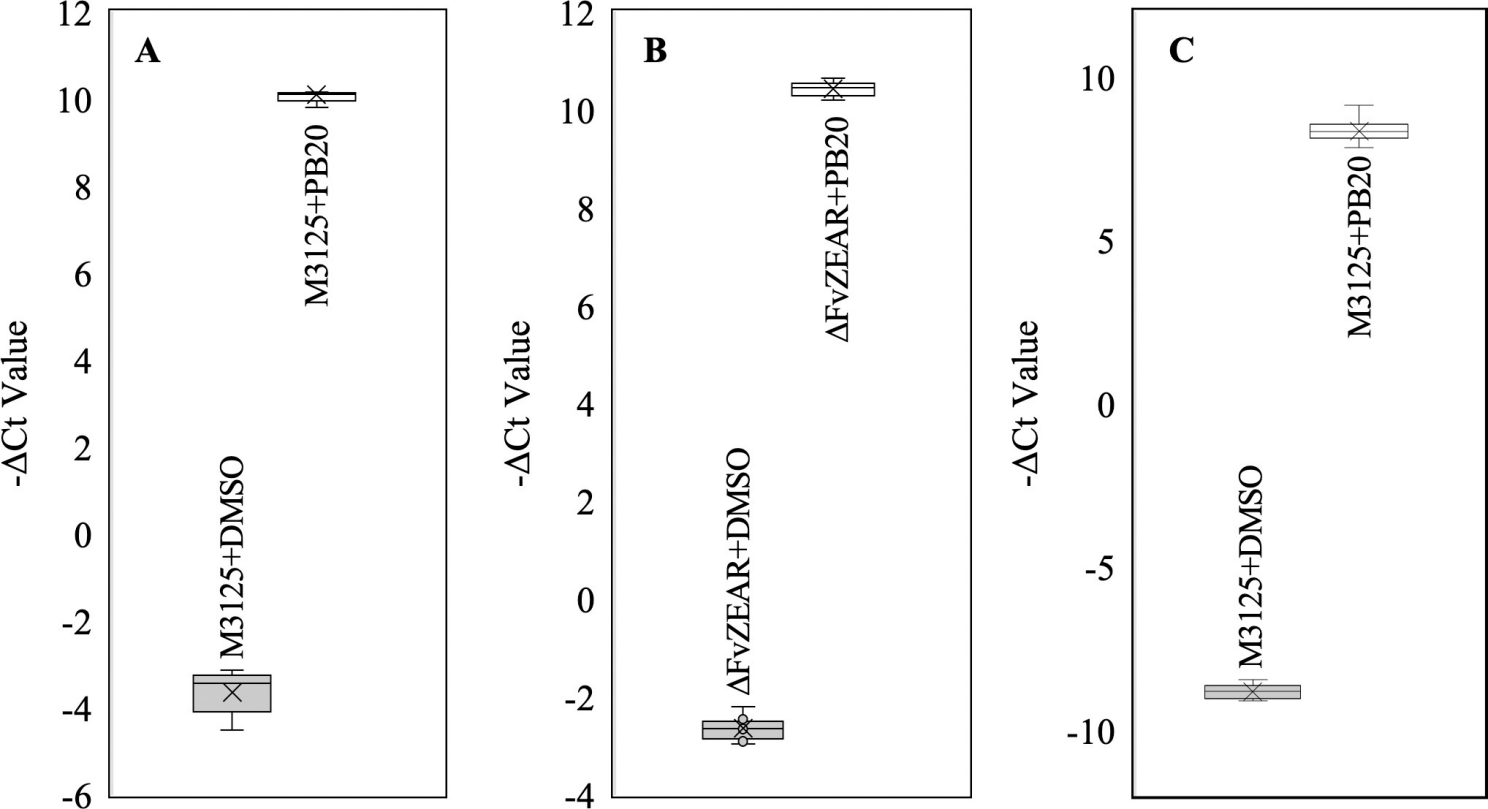
qRT-PCR results validated the pyrrocidine B induced up-regulation of *FvABC3* and *FvZBD1*, and that induction of *FvABC3* was independent of Δ*FvZEAR*. qRT-PCR was performed with M-3125 exposed or not exposed to 20 μg/mL pyrrocidine B for a final hour of growth after 47 hours in 2 mL of PDB. The Y-axis of the box plots shows the -ΔCt value, which was calculated by subtracting the Ct value of the β-tubulin reference gene from the Ct value of each gene of interest. Expression of *FvABC3* in (A) wild-type M-3125 and (B) the Δ*FvZEAR*-1 mutant. (C) Induction of *FvZBD1* in wild type upon exposure to pyrrocidine B. Three biological replicates, each with 3 technical replicates were assessed. All nine data points of each treatment are included in the box plots. The range of -ΔCt values is shown with error bars, and each box indicates first and third quartile. Mean and median are marked with an “×” and a line in each of the boxes.

**Fig 4:**
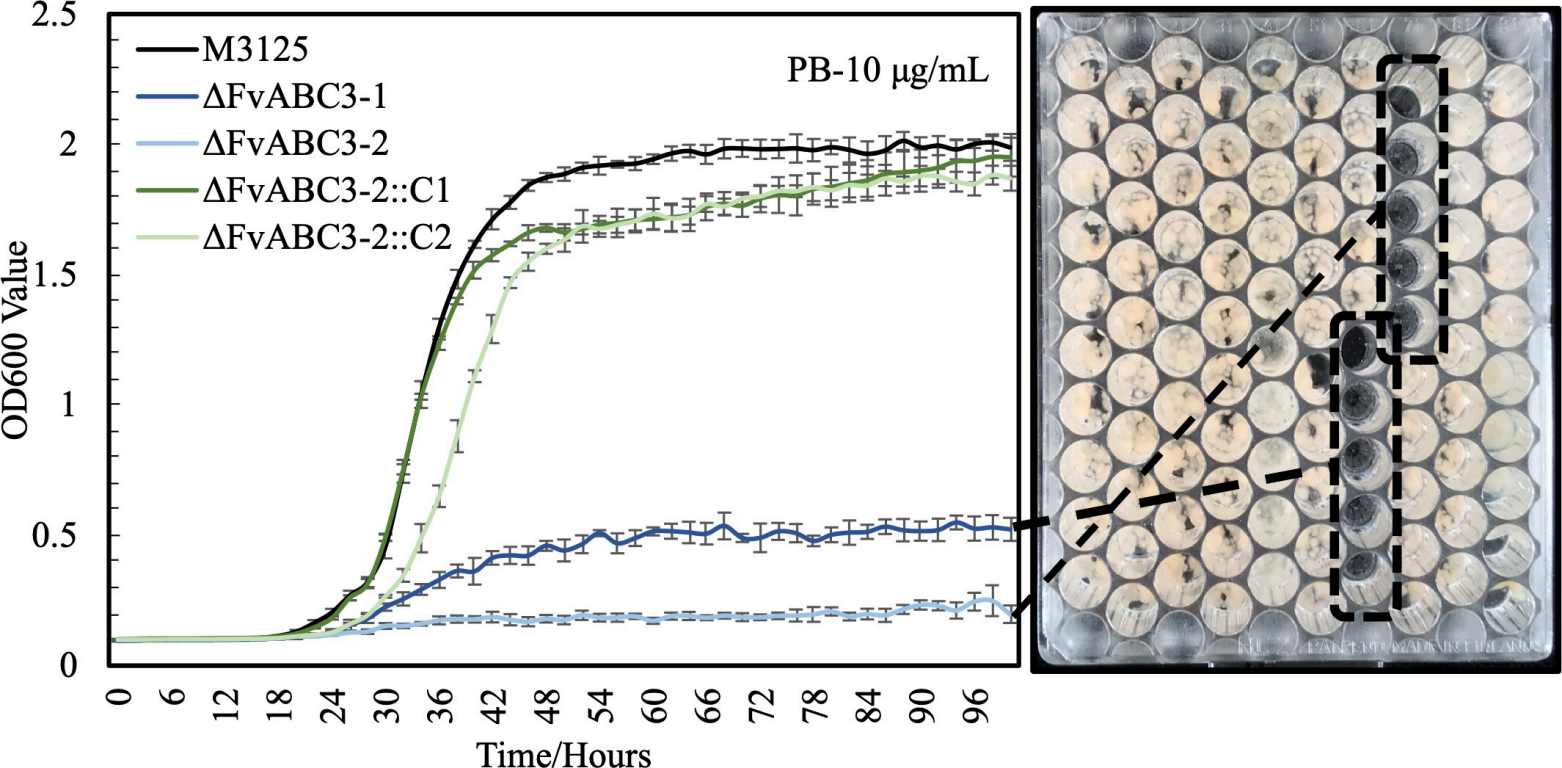
Deletion of *FvABC3* in *F*. *verticillioides* increased its sensitivity to pyrrocidine B. Strains were monitored for 100 hours in PDB medium amended with pyrrocidine B at 10 μg/mL. OD_600_ measurements taken every 2 hours were plotted (mean ± standard deviation). FRC M-3125 serves as the control (black curve). Two Δ*FvABC3* deletion mutants are shown in light and dark blue, and 2 complemented strains in light and dark green. Corresponding growth inhibition phenotypes of the Δ*FvABC3* deletion mutants are highlighted in the honeycomb plate after 100-hour incubation.

To investigate the significant role of this putative ABC transporter gene, a qRT-PCR assay was conducted to confirm the pyrrocidine B-induced expression levels of *FvABC3* and its adjacent transcription factor gene, *FvZEAR*. The *Fusarium graminearum* orthologs of both of these genes were previously characterized [17,18]. Both genes were highly induced in M-3125 PDB liquid cultures when exposed to pyrrocidine B at 20 μg/mL for 1 hour (Fig 3). The induction level of *FvABC3* remained comparably high when challenging the Δ*FvZEAR* mutant under the same conditions (Fig 3A and 3B). This suggested that pyrrocidine-induced *FvABC3* expression is independent of its neighboring transcription factor, which was consistent with the phenotype of no enhanced sensitivity to pyrrocidine B in Δ*FvZEAR* mutants, while *FvABC3* mutants showed extreme sensitivity (Fig 4). Evaluation of Δ*FvABC3* mutant strains revealed that the elevated sensitivity to pyrrocidine B was maintained even at 10 μg/mL, half the concentration utilized for transcriptional induction. Growth inhibition could be easily visualized in the microtiter culture plates after the 100-hour incubation with continuous shaking (Fig 4). The increased pyrrocidine B sensitivity of Δ*FvABC3* mutants was returned to a level similar to wild type by reintroducing the *FvABC3* gene into Δ*FvABC3* mutants by protoplast transformation.

### 5. Deletion of *FvABC3* did not alter fungal virulence on maize seedlings nor fumonisin production in GYAM medium

All the fungal strains tested in this experiment, including wild-type FRC M-3125, Δ*FvABC3* mutants, and the complemented strains, reduced maize seedling height by approximately 60% compared to uninoculated water control seedlings. Necrotic leaf lesions and other typical disease symptoms were observed across all fungal strains, and no change in virulence was observed between M-3125 and Δ*FvABC3* mutants (S3 Fig).

Since the Silver Queen maize cultivar used in the seedling assay is known to be highly sensitive to the phytotoxic effects of fumonisins, symptomology on that cultivar typically reflects the level of fumonisin production by the *F*. *verticillioides* strains tested [19,20]. To support previous seedling observations, fumonisin production capability of the Δ*FvABC3* mutants was assessed and compared to their wild-type progenitor in GYAM liquid cultures, a medium conducive to fumonisin production [21]. The fumonisin concentrations were normalized to the weight of vacuum-desiccated fungal tissue from the cultures. The individual gene deletion mutants did not exhibit significant differences in fumonisin B1 (FB1), B2 (FB2), or B3 (FB3) production. FRC M-3125 produced FB1 at an average concentration of 375 μg/mL/g, while two Δ*FvABC3* mutants, Δ*FvABC3*-1 and Δ*FvABC3*-2, produced 349 and 345 μg/mL/g, respectively (S4 Fig). As is typical, compared to FB1 production, all strains produced consistently less FB2 (69–75 μg/mL/g) and FB3 (152–172 μg/mL/g).

### 6. Deletion of *FvZBD1* caused a minor delay in growth under pyrrocidine B exposure

**Fig 5:**
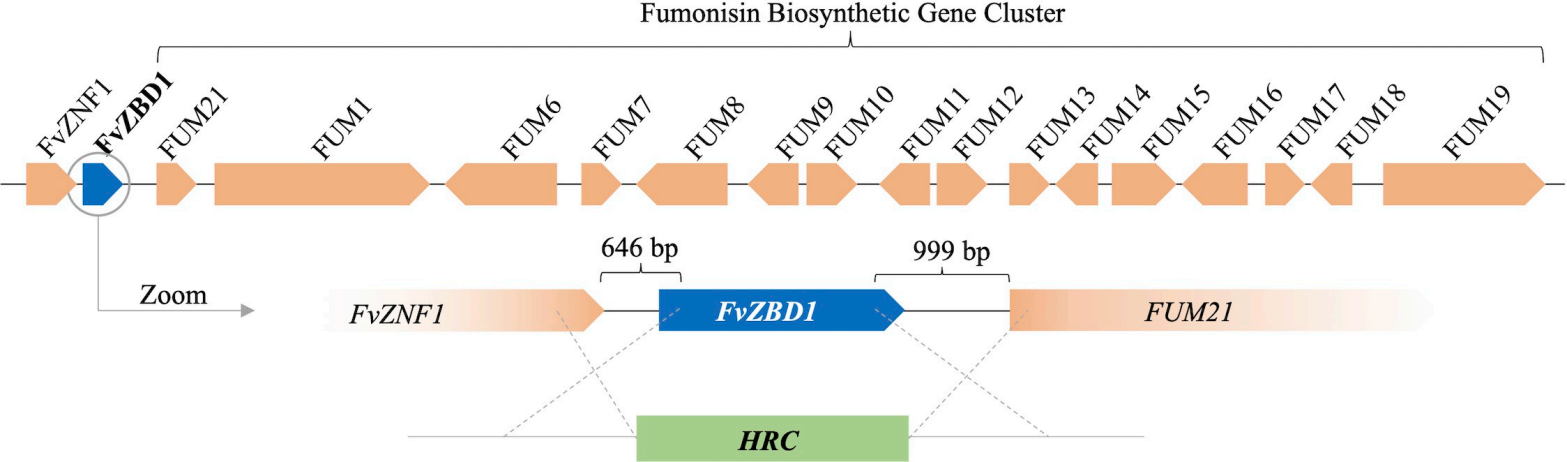
*FvZBD1* is adjacent to the well-characterized *FUM* cluster. Genes are represented by colored arrows with labels. The direction of arrows denotes the orientation of genes. A zoom-in section of the *FvZBD1* locus indicates the deletion occurs at 646 bp downstream of *FvZNF1* and 999 bp upstream of *FUM21* when generating Δ*FvZBD1* mutants. *FvZBD1* is replaced with the hygromycin resistance cassette (HRC).

*FvZBD1* (FVEG_00314) was the most highly induced gene from both the pyrrocidines A and B exposure treatments with a log2 induction value of 12 for each, which is approximately a 4100-fold change in expression (Table 1). We validated the induction of *FvZBD1* in M-3125 through qRT-PCR (Fig 3C). Interestingly, *FvZBD1* is directly adjacent to the fumonisin biosynthetic cluster (Fig 5). Due to the distinctive genomic location of *FvZBD1*, we further characterized phenotypic changes as a consequence of its deletion. When Δ*FvZBD1* mutants were challenged with 10 μg/mL pyrrocidine B, we observed a significant and repeatable delay in growth, compared to the wild type (S5 Fig). The phenotype of increased sensitivity was not observed in strains complemented in trans, suggesting that the lack of *FvZBD1* gene product is responsible for the mutant phenotypes, rather than any impact on the adjacent fumonisin biosynthetic cluster.

### 7. Deletion of *FvZBD1* significantly enhanced fungal virulence on maize seedlings

**Fig 6:**
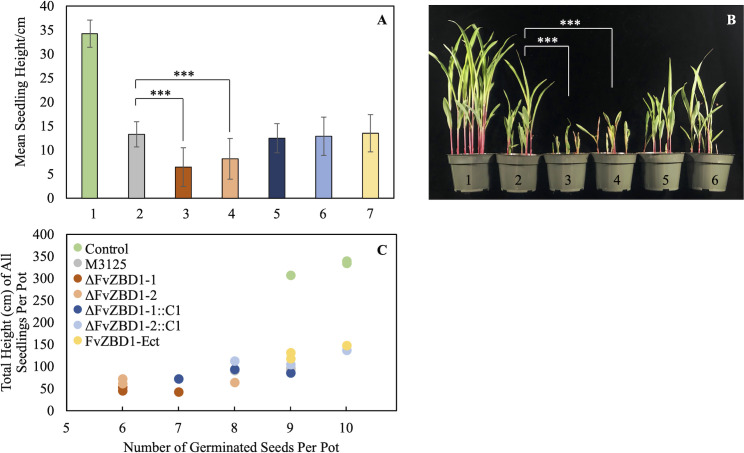
Deletion of *FvZBD1* in *F*. *verticillioides* significantly enhanced virulence on maize seedlings. Fifty Silver Queen maize seeds were inoculated with 10^4^/mL conidial suspensions for each of the different *F*. *verticillioides* strains prior to planting. Seeds treated with sterile water served as a control. Plants were grown for 14 days before measuring their heights and assessing numbers of germinated seeds. Three biological replicates were conducted. Results are shown from one representative trial. The other two trials showed the same overall trends. (**A**) Histogram showing the mean height of seedlings. Numbers on the X-axis correspond to the following treatments: **1**, sterile water control; **2**, M-3125; **3**, Δ*FvZBD1*-1; **4**, Δ*FvZBD1*-2; **5**, Δ*FvZBD1*-1::C-1; **6**, Δ*FvZBD1*-2::C-1; **7**, *FvZBD1*-Ect. Statistical analysis was conducted with the two-tailed Mann Whitney Wilcoxon test (***, p-value < 0.001). (**B**) Visualization of seedling growth among treatments. Numbers on the pots correspond to those in (A). (**C**) Two-dimensional visualization of seedling growth among different treatments. Each dot represents a technical replicate of a particular treatment. Total height (cm) of all seedlings per pot is denoted on the Y-axis, and the X-axis shows the number of germinated seeds per pot.

As expected, all the fungal treatments exhibited stunted growth of Silver Queen maize seedlings, compared to the uninoculated water control. However, Δ*FvZBD1* mutant-treated plants displayed the earliest onset of disease symptoms (on day 4 post planting, compared to day 6 for M-3125, not shown), increased disease severity with reduced growth, and higher frequency of necrotic lesions and other severe symptoms, compared to the wild-type treatment (Fig 6A and 6B). The mean heights of seedlings treated with Δ*FvZBD1*-1 and Δ*FvZBD1*-2 were 6.5 cm and 8.2 cm, respectively, whereas the mean heights of M-3125, ectopic, and complemented-strain treatments ranged from 12.5 cm to 13.5 cm. Two-tailed Mann Whitney Wilcoxon tests revealed significant differences in mean seedling heights between Δ*FvZBD1* mutant treatments and wild-type treatments, while there were no statistical distinctions between seedlings inoculated with wild type, ectopic, or complemented strains (Fig 6A). Uninoculated control plants showed the highest germination percentage and mean seedling height. Δ*FvZBD1* treatments showed the most stunted growth and the lowest germination percentage, while M-3125, ectopic, and complemented strain treatments exhibited better growth and greater germination (Fig 6B and 6C).

### 8. Enhanced seedling virulence of Δ*FvZBD1* mutants likely due to increased fumonisin production

**Fig 7:**
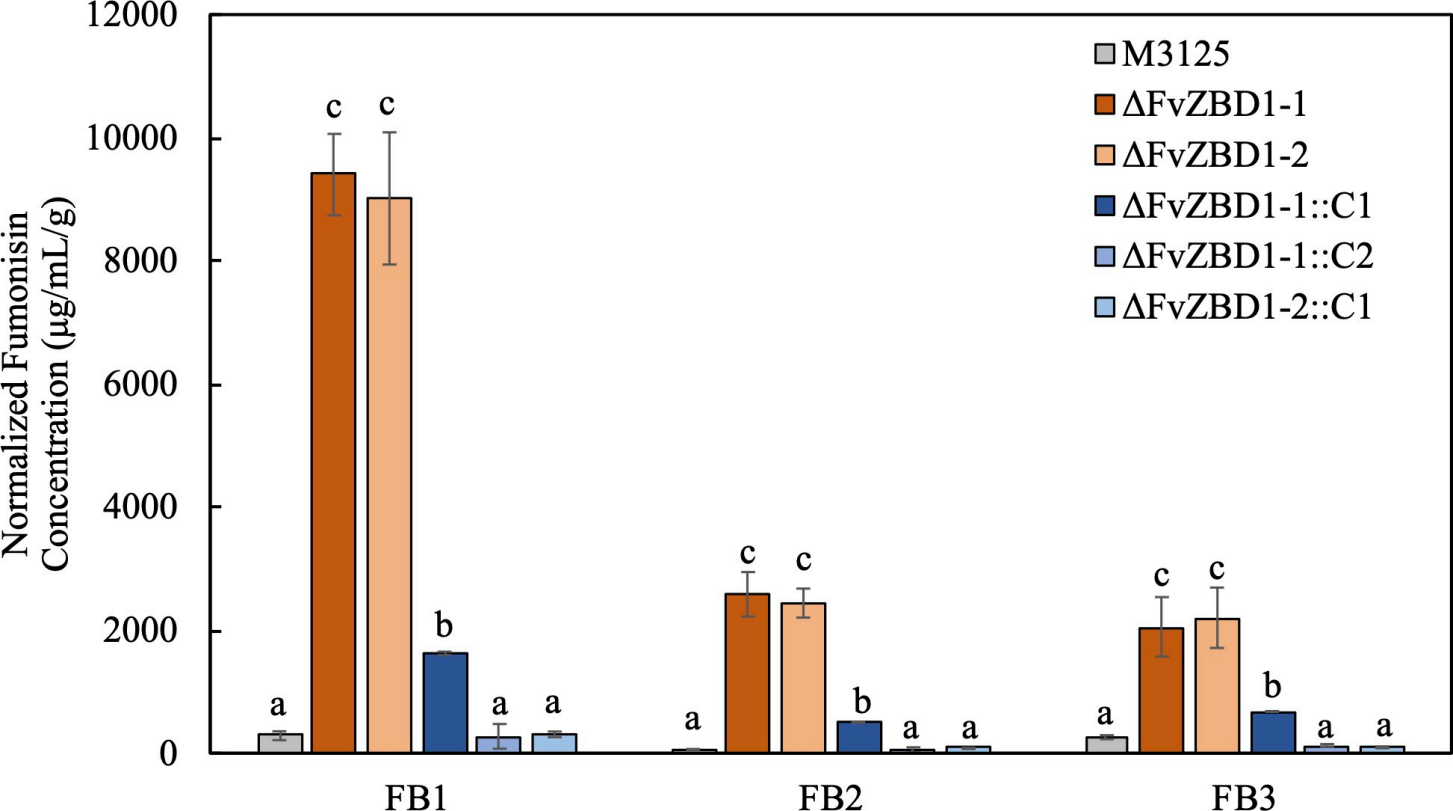
Deletion of *FvZBD1* dramatically increased fumonisin production in GYAM liquid cultures. Two milliliters of GYAM liquid medium in snap-cap tubes were inoculated with 10^4^ spores of each strain and cultured in the dark at 27°C, 250 rpm for 7 days. Fumonisin concentrations were determined by LC-MS and normalized to the vacuum-desiccated fungal mass weight, as indicated on the Y-axis. The experiment was conducted three times, with 3 technical replicates each. The two trials showed similar patterns, and one representative trial is plotted. Statistical differences (p-value < 0.05) were estimated with the two-tailed Mann Whitney Wilcoxon test and denoted with lower case letters for each fumonisin group. Strains sharing the same letters are not significantly different. FB1/FB2/FB3 represent fumonisin B1/B2/B3.

The enhanced seedling virulence of Δ*FvZBD1* mutants and the proximity of *FvZBD1* to the fumonisin biosynthetic cluster led us to evaluate fumonisin production by Δ*FvZBD1* mutants. Interestingly, deletion of *FvZBD1* elicited a remarkable increase in FB1, FB2, and FB3 production (Fig 7). The two Δ*FvZBD1* mutants produced over 9 mg/mL FB1 per gram of fungal tissue in GYAM liquid cultures, a > 30-fold increase compared to the 284.4 μg/mL/g for M-3125. The mean FB2 production of Δ*FvZBD1*-1 and Δ*FvZBD1*-2 mutants was 2581.3 and 2439.4 μg/mL/g, respectively, approximately a 40-fold increase over the 58.4 μg/mL/g in wild type. A prominent increase in FB3 production was also seen in Δ*FvZBD1* mutants. Δ*FvZBD1*-1 and Δ*FvZBD1*-2 mutants produced, on average, 2054.1 and 2200.7 μg/mL/g FB3, respectively, compared with wild-type M-3125 which produced an average of 260.1 μg/mL/g FB3 in GYAM liquid cultures. All *F*. *verticillioides* strains in this assay produced more FB1 than FB2 or FB3. M-3125 and complemented strains typically produced more FB3 than FB2, while Δ*FvZBD1* mutants exhibited an inverse trend by generating more FB2 than FB3.

### 9. Sub-inhibitory levels of pyrrocidine B nearly eliminate fumonisin production

**Fig 8:**
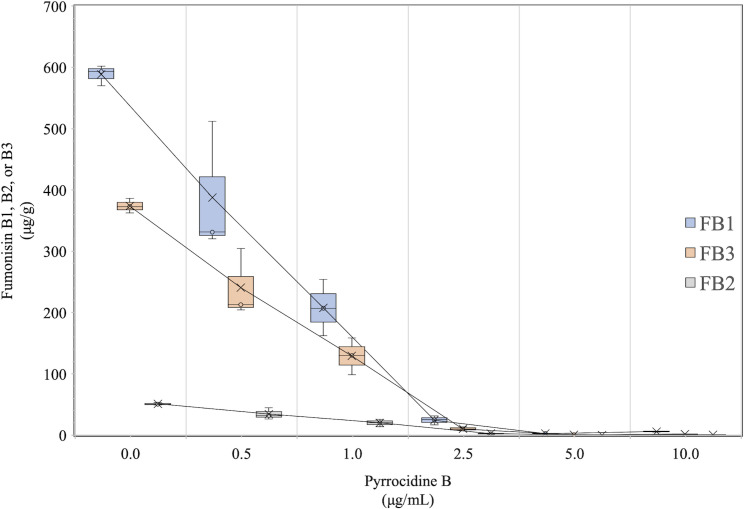
Pyrrocidine B reduces fumonisin production in a dose-dependent manner. Wild-type *Fusarium verticillioides* (FRC M-3125) was grown for 3 days in 3 mL potato dextrose broth (PDB) in a snap-cap, round bottom tube incubated at 27°C with shaking at 250 rpm. From this culture, 10 μL was inoculated into each of 21 replicate tubes containing 3 mL fresh PDB. These were incubated for 24 hrs as before, after which the following seven treatments were applied to triplicate cultures: No treatment control, DMSO control, 0.5, 1.0, 2.5, 5.0, and 10 μg/mL pyrrocidine B. The cultures were incubated as above for 4 days and then extracted and analyzed for FB1, FB2, and FB3. The experiment was conducted a minimum of three times, with similar results obtained from all experiments. Data from a single trial are presented.

Pyrrocidine B was very effective at reducing production of FB1, FB2, and FB3. Only 2.7 μg/g FB1 was detected in the treatment with 5 μg/mL pyrrocidine B, with essentially no FB2 or FB3 detected (Fig 8). This was a 99.5% reduction in FB1 production compared to the DMSO control. This was a sub-inhibitory dose of pyrrocidine B, thus this reduction was not due to overt antifungal effects on *F*. *verticillioides* growth. Consistent with the expression data and deletion of *FvZBD1*, pyrrocidine B appeared to repress fumonisin biosynthesis.

### 10. Δ*FvZBD1* mutants exhibited a more uniform growth morphology on GYAM plates compared to wild type

**Fig 9:**
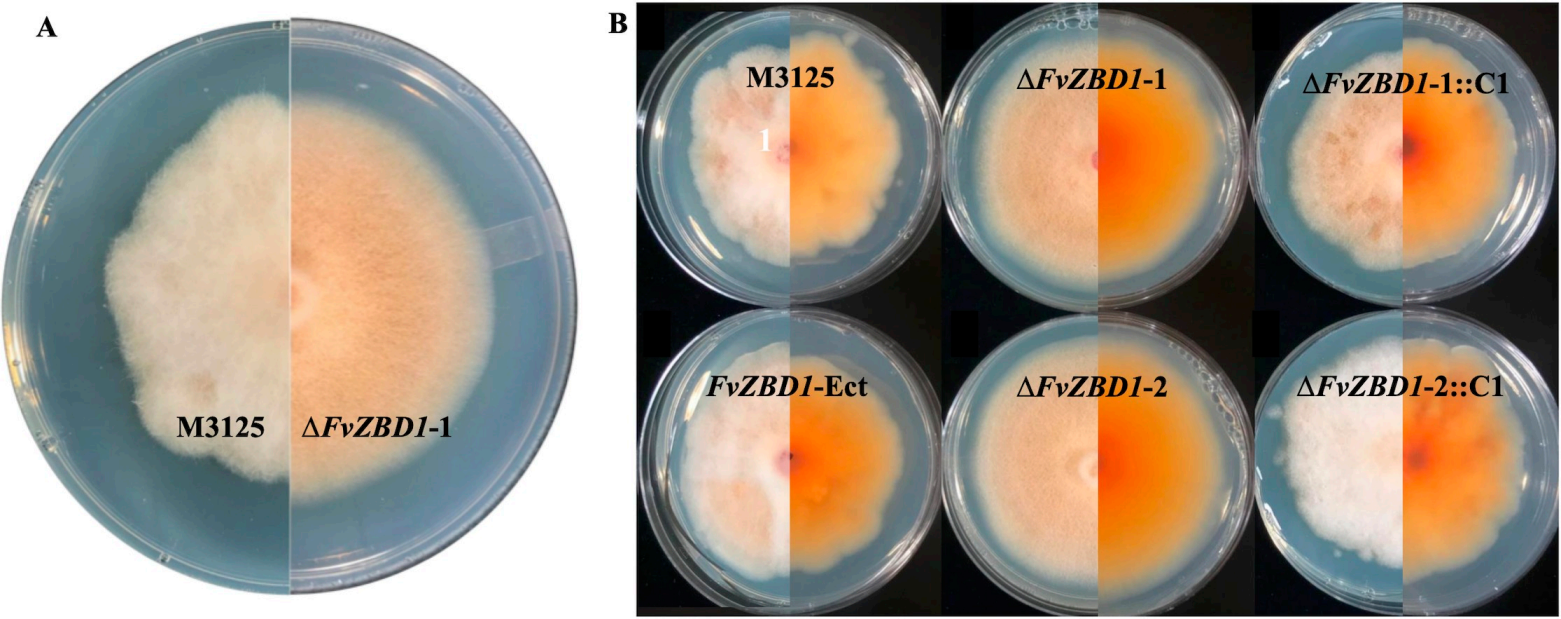
Δ*FvZBD1* mutants, but not wild type, displayed uniform colony margins on GYAM plates. **(A)** Seven-day-old GYAM agar cultures displayed different growth phenotypes of M-3125 (left) and Δ*FvZBD1*-1 (right), both shown from the front view. **(B)** Growth phenotypes of nine-day-old GYAM cultures for the *F*. *verticillioides* strains with both front and reverse views (left and right halves, respectively, of the split images). Genotypes are labeled as shown.

After 7-days incubation on GYAM plates, Δ*FvZBD1* mutants exhibited increased radial growth with smooth colony margins, while M-3125, an ectopic transformant, and complemented strains showed slightly slower growth with irregular, undulating colony edges and some sectoring (Fig 9A). Δ*FvZBD1* colonies appeared to be flat with enhanced orange pigmentation of the fungal mycelia, while M-3125 and complemented strains appeared less pigmented (Fig 9B). Rough colony margins were observed from 3 days after inoculation and became progressively more evident as colonies aged.

### 11. Pyrrocidines elicited the differential expression of lactamase-encoding genes

Pyrrocidine A displays higher toxicity than pyrrocidine B [8]. The only difference between these xenobiotic compounds is a double bond present in the lactam ring of pyrrocidine A, which implies the relevance of the lactam ring to antibiosis [8,9]. Inspired by our previous research on the molecular and functional characterization of fungal lactamases [12,22], we further investigated the transcriptional regulation of the 46 lactamase genes in the *F*. *verticillioides* genome. Four lactamase genes were up- or down-regulated by both pyrrocidine A and B treatments, four lactamase genes were exclusively induced by pyrrocidine A, and nine were exclusively regulated by pyrrocidine B (Table 2). Although FVEG_05734, FVEG_12457, and FVEG_13675, exhibited over 16-fold changes in gene expression, the average FPKM values remained low after induction at 23, 18, and 74, respectively, indicating their transcripts were not highly abundant despite dramatic induction.

**Table 2:** Lactamases differentially expressed in *Fusarium verticillioides* upon exposure to pyrrocidine A and/or B.

Gene#	Signal peptides	Pyrrocidine A Exposure		Pyrrocidine B Exposure	
		p-value^a^	Log2 FC^b^	p-value	Log2 FC
FVEG_03849	N	5.41E-67	1.52	0	3.77
FVEG_05734	N	1.55E+00	1.55	1.37E-57	4.05
FVEG_09854	N	2.42E-14	1.31	7.49E-15	1.30
FVEG_12637	N	2.44E-03	-2.08	4.89E-03	-1.78
FVEG_05685	N	1.50E-02	1.74	NS^**c**^	
FVEG_09433	N	3.90E-04	2.53	NS	
FVEG_12347	N	3.93E-04	1.45	NS	
FVEG_13172	N	3.56E-08	1.61	NS	
FVEG_05854	N	NS		3.60E-25	-3.15
FVEG_09904	N	NS		2.26E-14	-2.51
FVEG_10996	N	NS		2.27E-10	1.28
FVEG_12159	N	NS		2.09E-90	2.23
FVEG_12457	N	NS		1.88E-39	4.91
FVEG_12526	Y	NS		3.35E-02	1.37
FVEG_13253	N	NS		1.84E-03	-1.62
FVEG_13675	N	NS		4.47E-135	4.07
FVEG_16907	N	NS		1.14E-39	-2.97

^**a**^ p-value, false discovery rate-adjusted p-value

^**b**^ FC, fold change

^**c**^ NS, not significant

## Discussion

*F*. *verticillioides* and *S*. *zeae* frequently co-inhabit maize kernels, yet they partition their colonization to separate kernel tissues. The potential role of pyrrocidines in such partitioning and spatially limiting seed colonization by *F*. *verticillioides* inspired us to pursue the impact of these secondary metabolites on the *F*. *verticillioides* transcriptome. We aimed to gain insights into the survival mechanisms of *F*. *verticillioides* in the presence of inhibitory pyrrocidines by examining transcriptional responses to pyrrocidine exposure. RNA sequencing of the transcriptome revealed 770 and 4290 genes differentially responsive to pyrrocidine A and B, respectively. Of the 395 up-regulated genes shared by both treatments, we selected 10 gene targets for functional analyses based primarily on expression levels and significant fold changes. Preliminary screening of the single gene deletion mutants indicated that two genes, a putative ABC transporter gene FVEG_11089 (*FvABC3*) and a putative zinc-binding dehydrogenase gene FVEG_00314 (*FvZBD1*), proved to be particularly interesting and were characterized in greater detail. *FvABC3* was shown to have a crucial role in tolerating pyrrocidine B. Further, *FvZBD1*’s proximity to the well-characterized fumonisin biosynthetic gene cluster led us to assess the impact of *FvZBD1* on fumonisin production and virulence. To our surprise, deletion of *FvZBD1* significantly enhanced the fungal virulence on maize seedlings, which was correlated with the remarkable increase in fumonisin production by the mutants.

During the initial screening of deletion mutants with 20 μg/mL pyrrocidine B, only *ΔFvABC3* mutants exhibited significantly elevated sensitivity compared to wild type. Among the ten targeted genes, FVEG_17422 is another ABC transporter-encoding gene exhibiting induction upon exposure to both pyrrocidine A and B, with the level of induction being much higher in pyrrocidine B exposure than A (Table 1). However, deletion of FVEG_17422 did not result in a noticeable change in pyrrocidine B sensitivity in the initial screening with 20 μg/mL pyrrocidine B. Along with FVEG_11089 and FVEG_17422, FVEG_02410 and FVEG_16559 are another two ABC transporter encoding genes also exhibiting high induction levels (log2 fold change = 9.6 and 6.1, respectively) upon exposure to pyrrocidine B, and it would be interesting to investigate their impact on pyrrocidine B tolerance. It may be that despite the induction of these various ABC transporters, *FvABC3* may have functional specificity for pyrrocidines.

ABC transporters have been characterized in all extant phyla from prokaryotes to humans for their critical roles in drug resistance, which is also how some of them were first identified [23]. As the name indicates, ABC transporters mediate intake of the nutrients (importers; in prokaryotes only) or extrusion of toxins/drugs (exporters; in all phyla) across cellular membranes, which are energized by ATP binding and hydrolysis. A common characteristic of ABC transporters is that they possess nucleotide-binding domains (NBDs) and transmembrane domains (TMDs) responsible for substrate recognition and conformational changes [24]. *FvABC3* putatively encodes a protein of 1488 amino acids, and comparison with other sequenced strains it shares high protein sequence identities (> 85%) with clear orthologs in other *Fusarium* pathogens including *Fusarium fujikuoi*, *Fusarium proliferatum*, *F*. *graminearum*, *F*. *oxysporum*, and others. Similar to other fungal ABC transporters, FvABC3 topology contains two modules, each with a nucleotide-binding domain (NBD) and a transmembrane domain (TMD), reminiscent of its extensively studied *Saccharomyces cerevisiae* ortholog, PDR5 (S6 Fig). Similar to PDR5, the first NBD of FvABC3 is predicted at the N-terminus, followed by a TMD with five transmembrane segments (TMSs), and lastly another NBD and a TMD with 6 TMSs (S6A and S6B Fig). A close examination of the tertiary structures revealed conventional α-helical structures in the TMDs regions, while the NBDs possess two signature catalytic Walker A motifs or P-loops (GXXGXGKS/T) inferred by PSI-BLAST. The majority of conserved amino acids are predicted in the NBDs [25]. This conformation of FvABC3 resembles the crystal structure of a characterized multidrug resistance transporter (Protein Data Bank ID: 4F4C) in *Caenorhabditis elegans*, which is supportive of its role as a pyrrocidine extruder [25,26].

*FvABC3* is critical for resistance to pyrrocidine B. As a consequence of *FvABC3* deletion, mutant strains could not grow under exposure to pyrrocidine B at half the sub-inhibitory concentration used in RNA-Seq experimental treatments (Fig 4). Its ortholog in *F*. *graminearum*, *FgABC3* (FGSG_04580) was previously characterized for its role in azole fungicide tolerance [17], and deletion of *FgABC3* resulted in significantly reduced tolerance in the triazoles tebuconazole, prothioconazole, epoxyconazole, and fenarimol. Treatment of prothioconazole and fenarimol also elicited aberrant hyphal morphology in Δ*FgABC3* mutants. Interestingly, we assessed the growth rate of *ΔFvABC3* and *Δ*FVEG_17422 mutants as well as wild type on PDA plates amended with gradient concentrations of tebuconazole, and no growth differences were observed. This differential response to two structurally distinct xenobiotics suggests *FvABC3* may have substrate specificity with affinity to pyrrocidines but no utility for tolerance toward fungicides like tebuconazole in *F*. *verticillioides*.

A previously reported microarray based whole genome transcriptional analysis identified a zearalenone (ZEA) responsive gene, *ZEAR*, in *F*. *graminearum* with a 50-fold induction in expression upon exposure to zearalenone for one hour [18]. Its orthologous transcription factor encoding genes, *FvZEAR* (FVEG_11090) in *F*. *verticillioides* and *FoZEAR* in *F*. *oxysporum*, were also shown to be induced by ZEA exposure [18]. In our study, *FvZEAR* exhibited more than 39- and 174-fold induction upon exposure to pyrrocidine A and B, respectively. It is curious that *FvZEAR* is responsive to pyrrocidines and ZEA, despite their unrelated chemical structures. Deletion of *FvZEAR* did not impact the expression of the adjacent *FvABC3*, nor did it alter the sensitivity of *F*. *verticillioides* to pyrrocidine B. This implies the dispensable role of *FvZEAR* in tolerating pyrrocidine B. It is worth noting that FVEG_10488, encoding a putative transcription factor sharing 62% amino acid sequence identity with *FvZEAR* (FVEG_11090), was also significantly induced with log2 fold changes of 5.8 and 8.7 and FPKM values of 16 and 127 after pyrrocidine A and B exposure, respectively. Although the high fold changes were largely due to no observed expression of FVEG_10488 in the control treatment, it was still interesting to observe that this transcription factor was induced during pyrrocidine exposure. The amino acid similarity of FVEG_10488 to FVEG_11090, particularly the 80% identity over the first two-thirds of their alignment where the GAL4-like Zn2Cys6 binuclear cluster DNA-binding domain is located, may indicate possible functional redundancy regarding transcriptional regulation of genes such as *FvABC3*.

Fumonisins are polyketide-based mycotoxins produced by several closely related *Fusarium* species, and these metabolites, especially FB1, cause severe species-specific animal health problems [27,28]. Among the fumonisin producers, *F*. *verticillioides* has received worldwide attention due to its pathogenicity, toxicity, and wide occurrence on maize, a major economically important crop. Four different types of fumonisins are produced by *F*. *verticillioides* (FB1, FB2, FB3, and FB4), among which FB1 is the most abundant and toxic in naturally infected maize kernels [29]. Brown et al. (2012) described the co-regulated expression pattern of fumonisin biosynthetic (*FUM*) cluster genes with accession numbers ranging from FVEG_14633 (formerly FVEG_00315) to FVEG_00329. Interestingly, while the *FUM* genes displayed very low expression levels after pyrrocidine exposure (FPKM < 30), the putative zinc-binding dehydrogenase *FvZBD1* (FVEG_00314) immediately adjacent to the identified *FUM* cluster was induced to a high level (FPKM > 3400) in both pyrrocidine A and B treatments. In fact, *FvZBD1* was the most highly induced gene in these treatments. This induction of *FvZBD1* and suppression of *FUM* genes is consistent with the observed dose-response of decreasing fumonisin production with increasing pyrrocidine exposure. FB1 production was reduced 99.5% with exposure to just 5 μg/mL pyrrocidine B.

Inspired by its unique genomic position, we explored the impact of *FvZBD1* on fumonisin biosynthesis. Surprisingly, Δ*FvZBD1* strains showed dramatic elevation in production of fumonisins. Deletion of *FvZBD1* appeared to unleash the potential of fumonisin production in *F*. *verticillioides*, leading to > 30-fold increase in FB1 production, > 40-fold increase in FB2, and > 8-fold increase in FB3, compared to wild type. FB3 is a precursor to FB1 in the fumonisin biosynthetic pathway [30], and we observed a comparable amount of FB1 and FB3 production in M-3125 after 7-day incubation. Interestingly, deletion of *FvZBD1* favors the production of FB1 by more than four-fold over FB3 under the same growth conditions (Fig 7). The link between *FvZBD1* and the *FUM* cluster was not reflected in the previously published microarray data, where the expression profile of *FvZBD1* seemed unrelated to the production of fumonisins under normal growth conditions in GYAM medium [31]. Based on the premise of co-regulation, the *FvZBD1* gene does not appear to be a member of the *FUM* biosynthetic gene cluster, but since its deletion dramatically boosts fumonisin production, it may be that *FvZBD1* represents a noncanonical *FUM* cluster gene possibly involved in regulating production of the mycotoxin. While other *Fusarium* species possess ZBD1 orthologs, they may not produce fumonisin, such as is the case with *F*. *graminearum* or alternatively in some species that do produce fumonisin the *ZBD1* ortholog is not linked to the fumonisin biosynthetic cluster, as is the case of *F*. *fujikuoi*. However, in preliminary analysis, pyrrocidine B also dramatically inhibited synthesis of fumonisins in *F*. *fujikuoi*.

Along with the elevated production of fumonisins, we also observed enhanced virulence on maize seedlings, since the Silver Queen maize cultivar employed in this study is known to be highly sensitive to fumonisin phytotoxic effects [19,20]. Compared to wild-type and complemented-strain treatments, Δ*FvZBD1*-infected plants exhibited earlier appearance of disease symptoms, including poor germination rate, increased stunting and more severe lesion development leading to near whole-plant necrosis (Fig 6). The enhanced virulence of Δ*FvZBD1* mutants in the seedling assay reflects their elevated production of fumonisin, and to our knowledge, this is the first case of *F*. *verticillioides* exhibiting hypervirulence and hyperproduction of fumonisins due to a single-gene deletion. Such changes in physiology and pathology might be expected with the loss of a transcription factor (Cho et al., 2012), yet FvZBD1 has a conserved protein domain indicating its relationship to zinc-dependent alcohol dehydrogenases and quinone oxidoreductases, suggesting the encoded protein may impact metabolic activity and fumonisin production in a manner not yet understood but perhaps involving energy production or diversion of precursors.

As another example of enhanced activity, Δ*FvZBD1* mutants exhibited a more uniform and slightly faster growth morphology on fumonisin-conducive GYAM agar media (Fig 9). On the contrary, the wild-type, ectopic transformant, and *FvZBD1* complemented strains showed irregular and undulating colony edges, a type of morphology commonly observed when fungi are exposed to compounds that adversely affect growth. Thus, perhaps their growth morphology reflects reduced fitness in the presence of secondary metabolites being produced by the strains as a result of growth on GYAM. In comparison, these strains, including wild type, did not demonstrate undulating growth morphology on PDA, a medium not as conducive to fumonisin production. Considering the greatly enhanced fumonisin production in GYAM by Δ*FvZBD1* mutants and their healthier culture morphology, we may conclude that deletion of Δ*FvZBD1* not only unleashes the potential of fumonisin production in *F*. *verticillioides*, but there may also be enhanced fitness and tolerance to fumonisin and other metabolites in the GYAM environment.

Despite the importance of the lactam moiety in the pyrrocidines for toxicity, the *F*. *verticillioides* lactamase genes did not exhibit strong expression levels before and after pyrrocidine exposure (Table 2). For example, the average FPKM value for FVEG_03849 after pyrrocidine B exposure was 885 compared to 64 in the unexposed control. In comparison, the average FPKM values of *FvZBD1* were 0.37 in the control and 3768 after exposure to pyrrocidine B. Further, the induction FPKM values for the other lactamases were much lower than FVEG_03849. This may be an artifact of the experimental design of the exposure treatments. The RNA-Seq experiments were conducted with a one-hour induction by pyrrocidines A or B, and thus genes typically involved in early responses (e.g. transporters or metabolic genes) were among the most highly induced. In contrast, genes encoding lactamases and other hydrolytic enzymes may be involved at later time points. Therefore, transcriptional analysis of later time points may help identify hydrolytic lactamase-encoding genes involved in pyrrocidine B degradation.

**Fig 10:**
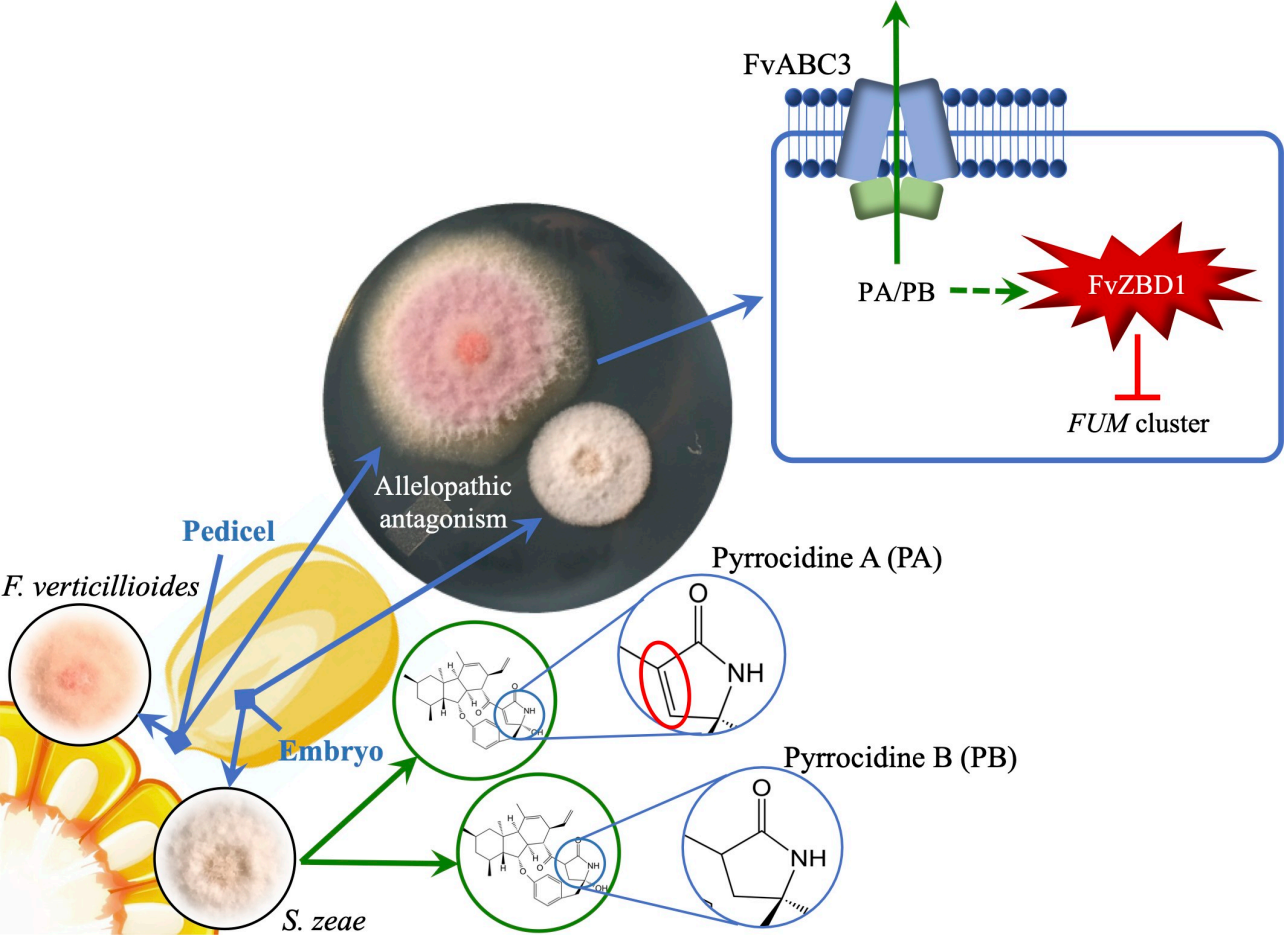
Illustration of biological antagonism between two maize seed endophytes, *F*. *verticillioides* and *S*. *zeae*. *F*. *verticillioides* is primarily confined to the pedicel of the maize kernel, while *S*. *zeae* is more frequently isolated from embryonic tissue. Pyrrocidine A and B, two lactam-containing antibiotics produced by *S*. *zeae*, are postulated to contribute to an allelopathic antagonism between the two fungi. The chemical structures of pyrrocidines differ by a double (red circle) or single bond within lactam ring, resulting in higher toxicity of pyrrocidine A than B. Detailed analyses were carried out for an ABC transporter encoding gene, *FvABC3*, and a putative zinc-binding dehydrogenase encoding gene, *FvZBD1*. We hypothesize that *FvABC3* facilitates persistence of *F*. *verticillioides* in maize seeds when encountering pyrrocidines, while the pyrrocidines induce the expression of *FvZBD1* in *F*. *verticillioides*, a gene negatively impacting the production of fumonisins and virulence in maize seedlings. *FvZBD1* is the most highly induced gene in both pyrrocidine A and B treatments, and its impact on fumonisin production provides evidence for allelochemical activity and response.

In summary, we identified large sets of genes differentially expressed upon pyrrocidine exposure and we functionally analyzed 10 pyrrocidine up-regulated genes for their role in conferring resistance to pyrrocidine B. Detailed analyses were carried out for an ABC transporter-encoding gene, *FvABC3* (FVEG_11089), required for wild type resistance to pyrrocidines, and a putative zinc-binding dehydrogenase encoding gene, *FvZBD1* (FVEG_00314), with a repressive impact on fumonisin production. We hypothesize that *FvABC3* facilitates persistence of *F*. *verticillioides* in maize seeds when encountering pyrrocidines, while the *S*. *zeae* pyrrocidines induced the expression of *FvZBD1* in *F*. *verticillioides*, a gene putatively having a negative impact on the production of fumonisins and virulence toward fumonisin sensitive maize seedlings (Fig 10). *FvZBD1* was the most highly induced gene in both pyrrocidine A and B treatments, and its impact on fumonisin production provides evidence for inter-fungus chemical signaling. We also propose to incorporate *FvZBD1* as a non-canonical part of the fumonisin biosynthetic cluster, since its deletion dramatically boosts fumonisin production. The strong induction of *FvZBD1* coupled with its genomic location and impact on fumonisin biosynthesis, combined with the negative effect of pyrrocidine B on fumonisin production, collectively provides substantial support for *S*. *zeae* as a potential biological control agent against *F*. *verticillioides* and fumonisin contamination of maize. Future experiments may investigate further the impact of *FvZBD1* on fumonisin production by over-expressing the gene or using controlled gene induction to assess the metabolic and transcriptomic impact on *F*. *verticillioides*.

## Materials and methods

### Fungal and bacterial strains, media and growth conditions

Strains of *F*. *verticillioides* used in this study are listed in Table 3. Wild-type strain FRC M-3125 was utilized for genetic characterization and modification. Fungal strains were routinely grown in a dark incubator at 27°C on potato dextrose agar (PDA; Neogen Food Safety, Lansing, MI, USA) or in potato dextrose broth (PDB; Neogen Food Safety, Lansing, MI, USA) at 250 rpm for 4 days. Transformed strains were screened on PDA plates amended with 150 μg/mL hygromycin B (Invitrogen, Carlsbad, CA, USA) or 300 μg/mL geneticin (Life Technologies, Carlsbad, CA, USA). Water agar (3%) plates were used for single conidial germling isolation [32].

**Table 3:** Fungal strains used in this study.

Index	Strain	Description and Source
HF1/1	FRC M-3125^**a**^	Wild-type strain used for mutant generation, also known as 7600 [13]
HF1/2	Δ*FvABC3*-1	FVEG_11089 deletion mutant in FRC M-3125
HF1/3	Δ*FvABC3*-2	FVEG_11089 deletion mutant in FRC M-3125
HF1/4	Δ*FvABC3*-2::C1	Δ*FvABC3*-2 deletion mutant complemented with FVEG_11089
HF1/5	Δ*FvABC3*-2::C2	Δ*FvABC3*-2 deletion mutant complemented with FVEG_11089
HF1/6	Δ*FvZEAR*-1	FVEG_11090 deletion mutant in FRC M-3125
HF1/8	Δ*FvZBD1*-1	FVEG_00314 deletion mutant in FRC M-3125
HF1/9	Δ*FvZBD1*-2	FVEG_00314 deletion mutant in FRC M-3125
HF1/10	Δ*FvZBD1*-1::C1	Δ*FvZBD1*-1 deletion mutant complemented with FVEG_00314
HF1/11	Δ*FvZBD1*-1::C2	Δ*FvZBD1*-1 deletion mutant complemented with FVEG_00314
HF1/12	Δ*FvZBD1*-2::C1	Δ*FvZBD1*-2 deletion mutant complemented with FVEG_00314
HF1/13	FvZBD-Ect	An ectopic strain containing HRC^**b**^ and intact ORF of FVEG_00314
HF1/14	ΔFv_08294	A transcription factor mutant with one known HRC genomic copy
HF1/15	ΔFv_01675	FVEG_01675 deletion mutant in FRC M-3125
HF1/16	ΔFv_07235	FVEG_07235 deletion mutant in FRC M-3125
HF1/17	ΔFv_09038	FVEG_09038 deletion mutant in FRC M-3125
HF1/18	ΔFv_13271	FVEG_13271 deletion mutant in FRC M-3125
HF1/19	ΔFv_13322	FVEG_13322 deletion mutant in FRC M-3125
HF1/20	ΔFv_17422	FVEG_17422 deletion mutant in FRC M-3125
HF1/21	ΔFv_17625	FVEG_17625 deletion mutant in FRC M-3125

^**a**^ FRC, Fusarium Research Center, Pennsylvania State University

^**b**^ HRC, hygromycin resistance cassette

To construct fungal gene deletion mutants, *Escherichia coli* (One Shot MAX Efficiency DH5αTM-T1R, Invitrogen, Carlsbad, CA, USA) was used as the recipient of OSCAR deletion constructs (see below) and was cultured in/on low sodium (0.5 g/L) Luria-Bertani (LB) medium amended with 100 μg/mL spectinomycin (Thermo Fisher Scientific, Waltham, MA, USA) at 37°C overnight. The fungal transformation was mediated by OSCAR deletion plasmid containing-*Agrobacterium tumefaciens* AGL-1 strains cultured on low sodium LB medium amended with 100 μg/mL spectinomycin at 27°C for 24–48 hours [15,16].

Growth curve analysis was performed initially with one confirmed deletion mutant per gene of interest with a Bioscreen C automated system (Growth Curves USA, Piscataway, NJ, USA). Fungal strains were inoculated in PDB (2×10^3^ conidia per 200 μL PDB per well) using Bioscreen honeycomb microtiter plates (Growth Curves Ab Ltd. Helsinki, Finland). Under these conditions *F*. *verticillioides* grows as a nearly pure culture of conidia, and thus OD reading are similar to those of yeast cultures. The wells were amended with 10 μg/mL pyrrocidine B dissolved in dimethyl sulfoxide (DMSO; Thermo Fisher Scientific, Waltham, MA, USA), and five replicates were prepared for each treatment. All treatments, including the controls, contained 0.5% DMSO. The microtiter plates were incubated 100 hours at 28°C with continuous shaking, and OD_600_ measurements were recorded every 30 min. For the presentation of the growth curves, only 2h incremental time points were plotted. The experiment was conducted three times. For genes FVEG_11089 and FVEG_00314 that showed growth defects in the presence of pyrrocidine B, additional Bioscreen experiments were carried out with at least two independently generated mutants and their complemented strains.

### Isolation and purification of pyrrocidine B

Pyrrocidine A was kindly gifted by the USDA, ARS, Mycotoxin Prevention and Applied Microbiology Research Unit (Peoria, IL, USA). Pyrrocidine B purification was conducted as previously described with minor modifications [7–9]. In brief, the ethyl acetate (E) extract from 12 lyophilized 15-day-old rice cultures (50 g of Kroger long grain white rice autoclaved with 50 mL deionized water in stationary 500 mL Erlenmeyer flasks) of *S*. *zeae* NRRL 13540 at 25°C in the dark was separated by flash chromatography utilizing silica powder (Sigma-Aldrich, St. Louis, MO, USA). The silica column was preconditioned with 100mL hexane (H) and eluted with 200 mL of 100%H, 80%H/20%E, 60%H/40%E, 40%H/60%E, 20%H/80%E, 100%E, 50%E/50%methanol (M), and 100%M. Each fraction was analyzed by LC-MS. Pyrrocidine B was detected in 40%H/60%E, 20%H/80%E, and 100%E fractions.

Fractions with pyrrocidines were combined, evaporated, re-dissolved in 200 mL hexane, and further purified using a 10g-Silica Sep-Pak cartridge (Waters Corporation, Milford, MA, USA). The Silica Sep-Pak cartridge was preconditioned with 100 mL H. Fractions were applied to the column and eluted with 100 mL of 100%H, 4 × 50 mL of 70%H/30%E, 2 × 50 mL of 60%H/40%E, 2 × 50 mL of 50%H/50%E, 100 mL of 30%H/70%E, 100mL 100%E, and 100 mL 100%M. Each fraction was analyzed by LC-MS. Pyrrocidine B was detected in the two 50%H/50%E fractions.

Fractions with pyrrocidines were combined, evaporated, and re-dissolved in 50 mL acetonitrile (A). Subsequently, 50 mL double distilled water (ddH_2_O) was added to obtain 100 mL volume. 10g-tC18 Sep-Pak cartridges (Waters Corporation) were used to further purify pyrrocidine B. The cartridge was conditioned with 200 mL of 100% methanol followed by 50%ddH_2_O/50%A. Samples were applied and eluted with 100 mL of 50%A/50%ddH_2_O, 2×50 mL of 60%A/40%ddH_2_O, 2×50 mL of 70%A/30%ddH_2_O, 2×50 mL of 80%A/20%ddH_2_O, 100 mL A, and 100mL E. Each fraction was analyzed by LC-MS. Pyrrocidine B was detected in 60%A/40%ddH_2_O and 70%A/30%ddH_2_O fractions. The subsequent steps remain the same as previously described [7–9]. The extraction process yielded 53.5 mg pyrrocidine B in the form of a fine light yellow powder.

### RNA-Seq experiment and data analyses

Two milliliter PDB cultures in sterile 14 mL round bottom snap-cap tubes, with caps unsnapped, were inoculated with 10^4^ M-3125 *F*. *verticillioides* conidia and grown at 27°C, 250 rpm for 47 hours, at which time 5 μL DMSO containing 10 μg or 40 μg (final concentrations of 5 μg/mL and 20 μg/mL) of pyrrocidine A and B, respectively, was added to the respective treatments. Five microliters of DMSO was added to negative control samples. All culture tubes were incubated for a final one-hour induction. Three biological replicates were prepared for each of the pyrrocidine A, pyrrocidine B, and negative control treatments. After the final hour of incubation (48 hours total), 1 mL liquid culture from each tube was prechilled on ice and pelleted by centrifugation at 8000 g for 5 min at 4°C, resuspended in 1 mL lysis buffer, and transferred to lysing matrix D tubes (MP Biomedicals, LLC, Santa Ana, CA, USA). The samples were homogenized with a FastPrep-24^TM^ 5G instrument (MP Biomedicals) at 6 m/s with 2 pulses of 30 s and a 1 min intervening pause at room temperature. Total RNA of each sample was isolated with a PureLink RNA Mini Kit (Thermo Fisher Scientific Inc., MA, USA) following the manufacturer’s protocol, and RNA quality was determined with an Agilent 2100 Bioanalyzer (Agilent Technologies, Palo Alto, CA, USA). Sequencing libraries were constructed with an Illumina Truseq DNA LT sample prep kit (Illumina Inc., San Diego, CA, USA) following the manufacturer’s protocol. Illumina library size validation was performed using the Agilent Tapestation 2200 High Sensitivity D1000 Assay (Part No. 5067–5584, Agilent Technologies, Santa Clara, CA, USA). Prior to equimolar library pool preparation, individual libraries were assayed for concentration by an Illumina library quantification kit (Product number KK4854, Kapa Biosystems, Inc, Wilmington, MA, USA) on a qPCR instrument (LightCycler 96, Roche Applied Science, Indianapolis, IN, USA). Each pool was clustered onboard an Illumina HiSeq2500 DNA sequencer with SR Rapid v2 flowcell clustering kits (Product number GD-402-4002, Illumina, San Diego, CA, USA). Single-end 50 bp sequencing was carried out with Rapid SBS v2 (Product number FC-402-4022, Illumina) reagents, and approximately 15 million reads were collected for each sample library. Sequencing reads were processed by *Cutadapt* 1.9.dev1 [33], *Trimmomatic* 0.32 [34] and custom scripts to remove adapters, low-quality reads, rRNA and organellar sequences, which were obtained from National Center for Biotechnology Information (NCBI) Gene Database (https://www.ncbi.nlm.nih.gov/). Reads were mapped to *F*. *verticillioides* 7600 genome by *Tophat* 2.0.13 [13,35], alignment sorted by *Samtools* 1.2 [36], and read count and expression estimation obtained by *HTseq* 0.6.1p1 [37] and *DESeq2* [38].

### Quantitative PCR confirmation of pyrrocidine B responsive genes

M-3125 PDB liquid culture preparation, pyrrocidine B challenge, and RNA extraction were conducted in the same way as described in RNA-Seq experiments. Three biological replicates were prepared for treatment groups and the control. For qRT-PCR, RNA samples were digested with DNase (Turbo DNA-freeTM Kit, Thermo Fisher Scientific) following the manufacturer’s protocol and analyzed with an Agilent 2100 Bioanalyzer to control quality (RNA-integrity number > 8). Conventional PCR (primers P1/50 and P1/51) targeting the β-tubulin reference gene (FVEG_04081) was performed with DNase digested RNA templates, with standard *Taq* based PCR followed by electrophoresis to ensure samples were free of DNA contamination. qPCR reactions were carried out using a one-step qRT-PCR Kit (SuperScript III Platinum SYBR Green One-Step qRT-PCR Kit, Thermo Fisher Scientific) with 3 technical replicates for each biological replicate following the manufacturer’s protocol. The data were normalized to the expression level of the β-tubulin gene (primers P1/50 and P1/51) by subtracting the β-tubulin Ct values from the Ct values of the genes of interest. The real-time primers used in this study are shown in Table 4.

**Table 4:** Primers used in this study.

Index	Primer Name	Primer Sequence (5' to 3')	Description
HP1/1	FVEG_11089_O1	GGGGACAGCTTTCTTGTACAAAGTGGAA AATCTTGAGCTGAGAGATCATAC	Amplification of 5' flank of *FvABC3* Amplification of 5' flank of *FvABC3*
HP1/2	FVEG_11089_O2	GGGGACTGCTTTTTTGTACAAACTTGT GTACCTGGTTAACTCTGTAAACT	
HP1/3	FVEG_11089_O3	GGGGACAACTTTGTATAGAAAAGTTGTT GAGAAGTACTATAGACTGGTTTGG	Amplification of 3' flank of *FvABC3* Amplification of 3' flank of *FvABC3*
HP1/4	FVEG_11089_O4	GGGGACAACTTTGTATAATAAAGTTGT GTCATGAAGATGGCTAAGATTTG	
HP1/5	FVEG_11089_ORF_F	CTTGGTTGATATGCCGTATAAGA	Confirmation of *FvABC3* ORF Confirmation of *FvABC3* ORF
HP1/6	FVEG_11089_ORF_R	GAATACTACCTACAGTGGAACAAG	
HP1/7	FVEG_11089_5’_out	CGGAGTTTCAAAAGATTGCTAATC	Confirmation of the integrity of outer sequences flanking the 5' flank of *FvABC3*
HP1/8	FVEG_11089_3’_out	GAGAGTTTATCGGTTGTGTATTGG	Confirmation of the integrity of outer sequences flanking the 3' flank of *FvABC3*
HP1/9	FVEG_11089_RT_F	ATCTTGCCTCAGCCATTCTATC	Amplification of *FvABC3* ORF for qPCR and qRT PCR Amplification of *FvABC3* ORF for qPCR and qRT PCR
HP1/10	FVEG_11089_RT_R	CAGGTTGTGCCGTTGAGA	
HP1/11	FVEG_11090_O1	GGGGACAGCTTTCTTGTACAAAGTGGAAGACCAAAGAGTTCAGACTGTAAG	Amplification of 5' flank of *FvZEAR* Amplification of 5' flank of *FvZEAR*
HP1/12	FVEG_11090_O2	GGGGACTGCTTTTTTGTACAAACTTGTCGAATCGAAATGCAGAGAATAATAG	
HP1/13	FVEG_11090_O3	GGGGACAACTTTGTATAGAAAAGTTGTTGGCATATAGCTGTGAATCACTAA	Amplification of 3' flank of *FvZEAR* Amplification of 3' flank of *FvZEAR*
HP1/14	FVEG_11090_O4	GGGGACAACTTTGTATAATAAAGTTGTTAGTACAAGGGATGAAGAGAAATG	
HP1/15	FVEG_11090_ORF_F	GAAGAAATGTGACAGAAAAAGTCC	Confirmation of *FvZEAR* ORF Confirmation of *FvZEAR* ORF
HP1/16	FVEG_11090_ORF_R	ATCCATTCAGTTTTACTCATACCG	
HP1/17	FVEG_11090_5’_out	TAGAGACTCTTGTAGGTCTCAATC	Confirmation of the integrity of outer sequences flanking the 5' flank of *FvZEAR*
HP1/18	FVEG_11090_3’_out	CGATGTAGGCGTTACTAGTTTTAG	Confirmation of the integrity of outer sequences flanking the 3' flank of *FvZEAR*
HP1/19	FVEG_11090_RT_F	TATCCCGAGTACACCTGCT	Amplification of *FvZEAR* ORF for qPCR and qRT PCR Amplification of *FvZEAR* ORF for qPCR and qRT PCR
HP1/20	FVEG_11090_RT_R	CTCTTCACACCTCTCCAATCTC	
HP1/21	FVEG_00314_O1	GGGGACAGCTTTCTTGTACAAAGTGGAAGAAGAAACCGAAAGTCACAAATG	Amplification of 5' flank of *FvZBD1* Amplification of 5' flank of *FvZBD1*
HP1/22	FVEG_00314_O2	GGGGACTGCTTTTTTGTACAAACTTGTGGTATATGTCAGTCAACCAGAAG	
HP1/23	FVEG_00314_O3	GGGGACAACTTTGTATAGAAAAGTTGTTACTTCAGAAGCGTAGGATTATAG	Amplification of 3' flank of *FvZBD1* Amplification of 3' flank of *FvZBD1*
HP1/24	FVEG_00314_O4	GGGGACAACTTTGTATAATAAAGTTGTCGTCGAAACATTGAGAGATAAAC	
HP1/25	FVEG_00314_ORF_F	GAGTTTGAGAAACCTCATCTCTC	Confirmation of *FvZBD1* ORF Confirmation of *FvZBD1* ORF
HP1/26	FVEG_00314_ORF_R	TACCAGATCTGCAAGAAATCAAG	
HP1/27	FVEG_00314_5’_out	CATTTTCCATCTTTCACAAACCTC	Confirmation of the integrity of outer sequences flanking the 5' flank of *FvZBD1*
HP1/28	FVEG_00314_3’_out	CTCTCCAATAATACTCGAACACTG	Confirmation of the integrity of outer sequences flanking the 3' flank of *FvZBD1*
P1/3	Hyg_For_out	AGAGCTTGGTTGACGGCAATTTCG	Confirmation of the integrity of outer sequences flanking the 3' flank of target gene
P1/4	Hyg_Rev_out	GCCGATGCAAAGTGCCGATAAACA	Confirmation of the integrity of outer sequences flanking the 5' flank of target gene
P1/46	HygMarker_F	GACAGGAACGAGGACATTATTA	Confirmation of HRC ORF Confirmation of HRC ORF
P1/47	HygMarker_R	GCTCTGATAGAGTTGGTCAAG	
P1/52	qPCR_Hyg_for	TCGATGAGCTGATGCTTTG	Amplification of HRC ORF for qPCR and qRT PCR Amplification of HRC ORF for qPCR and qRT PCR
P1/53	qPCR_Hyg_rev	GTTGGCGACCTCGTATTG	
P1/50	TUB2-F	CAGCGTTCCTGAGTTGACCCAACAG	Amplification of β-tubulin ORF for qPCR and qRT PCR Amplification of β-tubulin ORF for qPCR and qRT PCR
P1/51	TUB2-R	CTGGACGTTGCGCATCTGATCCTCG	
P10/27	FVEG_00314_RT_F	GGTCTCAGGAGGATTGCTAAAG	Amplification of FVEG_00314 ORF for qRT PCR
P10/28	FVEG_00314_RT_R	GCACGATACTGAACCAGAGATAG	Amplification of FVEG_00314 ORF for qRT PCR
P10/29	FVEG_01675 qPCR F	CTGGTGATCATCTGGCTTT	Amplification of FVEG_01675 ORF for qRT PCR
P10/30	FVEG_01675 qPCR R	AATCTTGCCACTTCCTCTG	Amplification of FVEG_01675 ORF for qRT PCR
P10/31	FVEG_07235 qPCR F	ACCAATACTTCAACGCACTC	Amplification of FVEG_07235 ORF for qRT PCR
P10/32	FVEG_07235 qPCR R	GATTGTAGCCTTCCCACTTAAA	Amplification of FVEG_07235 ORF for qRT PCR
P10/33	FVEG_09038 qPCR F	CTCATCTTGTCAGCACTAGC	Amplification of FVEG_09038 ORF for qRT PCR
P10/34	FVEG_09038 qPCR R	GCTTAGGAACCACACCATC	Amplification of FVEG_09038 ORF for qRT PCR
P10/35	FVEG_13271 qPCR F	CCAGGCGTCTTTACAGATTAC	Amplification of FVEG_13271 ORF for qRT PCR
P10/36	FVEG_13271 qPCR R	GAACAGTGGTCAAGGTGATT	Amplification of FVEG_13271 ORF for qRT PCR
P10/37	FVEG_13322 qPCR F	CGAAGTGGATGGCAATGAG	Amplification of FVEG_13322 ORF for qRT PCR
P10/38	FVEG_13322 qPCR R	AGAGACAGCAAACGCAATAA	Amplification of FVEG_13322 ORF for qRT PCR
P10/39	FVEG_17422 qPCR F	CGTCGATTGTCACTCTCTTG	Amplification of FVEG_17422 ORF for qRT PCR
P10/40	FVEG_17422 qPCR R	CCACCCAGTTCGGTAGATA	Amplification of FVEG_17422 ORF for qRT PCR
P10/41	FVEG_17625 qPCR F	GCGCTCGGATATCTGGT	Amplification of FVEG_17625 ORF for qRT PCR
P10/42	FVEG_17625 qPCR R	GTTGGCGTGCTCTGAAA	Amplification of FVEG_17625 ORF for qRT PCR

### Gene deletion and complementation

Ten select up-regulated genes identified from the RNA-Seq experiment were targeted to generate single deletion mutants. Plasmids pMG00314_OSCAR, pMG01675_OSCAR, pMG07235_OSCAR, pMG09038_OSCAR, pMG11089_OSCAR, pMG11090_OSCAR, pMG13271_OSCAR, pMG13322_OSCAR, pMG17422_OSCAR, and pMG17625_OSCAR were created as the gene deletion constructs for FVEG_00314 (*FvZBD1*), FVEG_01675, FVEG_07235, FVEG_09038, FVEG_11089 (*FvABC3*), FVEG_11090 (*FvZEAR*), FVEG_13271, FVEG_13322, FVEG_17422, and FVEG_17625, respectively, using the OSCAR method [15,16]. Primers used in this study are listed in Table 4 and S2 Table. Detailed steps are described below for *FvABC3*, *FvZEAR*, and *FvZBD1*. Two sets of primers were synthesized to amplify 1kb of the 5’ flank sequence (primers HP1/1 and HP1/2 for *FvABC3*; HP1/11 and HP1/12 for *FvZEAR*; HP1/21 and HP1/22 for *FvZBD1*) and the 3’ flank sequence (primers HP1/3 and HP1/4 for *FvABC3*; HP1/13 and HP1/14 for *FvZEAR*; HP1/23 and HP1/24 for *FvZBD1*) of the target open reading frames (ORFs) to generate constructs including the surrounding flanks but excluding the ORF. Each gene-encoding region was deleted in M-3125 through *Agrobacterium tumefaciens*-mediated transformation and double crossover homologous recombination. Hygromycin-resistant transformants were screened for the presence of the HRC (primer P1/46 and P1/47) and absence of the corresponding ORFs (primer pairs: HP1/5 and HP1/6 for *FvABC3*; HP1/15 and HP1/16 for *FvZEAR*; HP1/25 and HP1/26 for *FvZBD1*). Transformant candidates were single-spore isolated and confirmed by PCR screening subjected to the following criteria: 1) presence of the HRC; 2) absence of corresponding ORFs; 3) homologous recombination at the 5’ flank and 4) homologous recombination at the 3’ flank of the target gene with expected sizes (*FvABC3* 5’ flank, HP1/7 and P1/4; *FvABC3* 3’ flank HP1/8 and P1/3; *FvZEAR* 5’ flank, HP1/17 and P1/3; *FvZEAR* 3’ flank, HP1/18 and P1/4; *FvZBD1* 5’ flank, HP1/27 and P1/3; *FvZBD1* 3’ flank, HP1/28 and P1/4). To confirm single insertion events the copy number of HRC was determined by qPCR (see below). Similar gene deletion methods were also applied to the other seven targeted genes, and corresponding primers are listed in S2 Table.

For genetic complementation of mutants, the wild-type *FvABC3*, *FvZEAR*, and *FvZBD1* genes were amplified with primer pairs HP1/1+HP1/4, HP1/11+HP1/14, and HP1/21+HP1/24, respectively, to encompass the ORF regions plus 5’ and 3’ flanks, each of 1kb in length. The reactions were prepared with TaKaRa high fidelity Taq (TaKaRa Bio, Kyoto, Japan) following the manufacturer’s protocol. The amplicons were purified (QIAquick PCR purification kit, Qiagen, Inc., Valencia, CA, USA) and combined with undigested pGEN-NotI (1 μg) for PEG-mediated co-transformation of protoplasts [39] generated from the Δ*FvABC3*, Δ*FvZEAR*, Δ*FvZBD1* deletion strains. Transformants were selected on geneticin (300 μg/mL; Sigma-Aldrich) and screened by ORF primer pairs HP1/5+HP1/6, HP1/15+HP1/16, and HP1/25+HP1/26 to confirm complementation fragment integration of *FvABC3*, *FvZEAR*, and *FvZBD1* respectively. PCR reactions were carried out with NEB Taq 2X Master Mix (New England Biolabs, Ipswich, Massachusetts, USA) following the manufacturer’s protocol. Ectopic insertion of the complementing fragment was confirmed by retention of hygromycin resistance in all complemented strains reported here.

### Copy number determination

To ensure only one copy of HRC was introduced into the deletion mutant genomes, we further determined the copy number of HRC in deletion mutants. Genomic DNA was extracted from each 4-day old 50 mL PDB culture using the DNeasy Plant Mini Kit (Qiagen, Inc.) following the manufacturer’s protocol. M-3125 served as the negative control, and strain ΔFv_08294 (known single HRC copy) served as the positive control. Quantitative PCR was performed using Platinum Taq DNA Polymerase (Thermo Fisher Scientific) and SYBR Green I dye (Thermo Fisher Scientific) following the manufacturer’s protocol, using the DNeasy extracted genomic DNA. The relative copy number of target genes was normalized to the single copy reference β-tubulin (FVEG_04081) gene (primers P1/50 and P1/51), and calculated via the 2^−*ΔΔCt*^ method [40].

### MIPS functional enrichment analysis

Functional enrichment analysis was conducted with MIPS Functional Catalogue (FunCat) web server with *Fusarium verticillioides* 7600 –p3_p15553_Fus_verti_v31 serving as the reference species database [14]. A p-value of 0.05 was applied to filter the enrichment results.

### Maize seedling assay

Seeds of sweet corn variety Silver Queen (W. Atlee Burpee & Co., Warminster, PA, USA) were treated as previously described to eliminate pre-existent endophytes and surface microbes [19]. Approximately 50 seeds were placed in a 100-mm Petri dish and immersed in 10 mL sterile deionized water (SDW) containing 10^5^ conidia. Ten milliliters of SDW was added to the uninoculated control. After 16-hour incubation in the dark at 27°C, 10 seeds were planted in one 4-in azalea pot filled with twice-autoclaved moist Fafard 2 potting mix (Agawam, MA, USA). Three pots per treatment were prepared, situated on sterile plastic saucers in plastic trays, and watered from below by filling the saucers on days 0, 2, 4, and 6. Subsequent watering was performed as needed and also added from below. Plants were incubated for 14 days in a growth room alternating with 16 hours daylight (approximately 320 μmol/m^2^/s) at 30°C and 8 hours in dark at 20°C. On day 14, plants were harvested by cutting at the first node at the soil surface, and corresponding heights were measured for each seedling. Disease symptoms were visually inspected, and the experiment was repeated two times.

### Fumonisin production assay

Fumonisin production assays were conducted in GYAM medium, containing 0.05% yeast extract, 0.24 M glucose, 8 mM L-asparagine, 1.7 mM NaCl, 4.4 mM K_2_HPO_4_, 2 mM MgSO_4_, 8.8 mM CaCl_2_, and 5.0 mM malic acid [41]. Two milliliters of GYAM medium in sterile 14 mL snap-cap culture tubes, with caps unsnapped, were inoculated with 10^4^ spores and cultured in the dark at 27°C, 250 rpm for 7 days. To extract the whole culture, 2 mL of acetonitrile + 5% formic acid was added to each culture tube, which stood at room temperature for 3 hours after mixing thoroughly. The mixtures were vacuum-filtered through sterile 25 mm, 5-micron pore size MAGNA nylon filters (N50SP2500, MSI, Westboro, MA), which were previously desiccated under vacuum at room temperature for > 24 hours prior to obtaining the tare weight. The mycelial mass collected on the filter was desiccated under vacuum for > 24 hours before weighing. The filtered extract (roughly 3 mL) was diluted (1:1) by adding 3 mL ddH_2_O. The diluted samples were analyzed as previously described using LC-MS [42].

### Pyrrocidine B exposure and impact on fumonisin production

Wild-type *Fusarium verticillioides* (FRC M-3125) was grown for 3 days in 3 mL PDB in unsnapped 14 mL snap-cap tubes incubated at 27°C with shaking at 250 rpm. From this culture, 10 μL was inoculated into each of 21 replicate tubes containing 3 mL fresh PDB to create identical synchronized cultures. These were incubated for 24 hrs as before, after which the following seven treatments were applied to triplicate cultures: 1) No treatment control; 2) DMSO control (30 μL DMSO); 3) 0.5 μg/mL pyrrocidine B (1.5 μL 1 mg/mL pyrrocidine B in DMSO plus 28.5 μL DMSO); 4) 1.0 μg/mL pyrrocidine B (3.0 μL 1 mg/mL pyrrocidine B in DMSO plus 27.0 μL DMSO); 5) 2.5 μg/mL pyrrocidine B (7.5 μL 1 mg/mL pyrrocidine B in DMSO plus 22.5 μL DMSO); 6) 5.0 μg/mL pyrrocidine B (15.0 μL 1 mg/mL pyrrocidine B in DMSO plus 15.0 μL DMSO); 7) 10.0 μg/ml pyrrocidine B (30.0 μL 1 mg/mL pyrrocidine B in DMSO). These 21 cultures were incubated as above for 4 days and then stored at 4°C as needed (no more than 24 hrs) until ready for processing. Nylon filters (Micron Separations Inc, 5 μm) were dried under vacuum in a desiccator and tare weights were obtained. Three mL of acetonitrile +5% formic acid (MP Biomedicals, ≥ 99.9%; Mallinckrodt, 88%) were added to each culture tube and mixed thoroughly. The cultures were filtered using the dried nylon filters, and the filters plus fungal culture were once again dried and weighed to obtain fungal dry weight. The filtrate was diluted as necessary to be analyzed by HPLC-MS (Dionex ultimate 3000; Thermo LTQ-XL). An Almtakt Cadenza CW-C18 column (150 mm x 2 mm, 3 μm) provided the separation using a 0.2 mL/min gradient elution, 30% acetonitrile +0.1% formic acid (MP Biomedicals, ≥ 99.9%; Fluka, 98%) brought to 100% over 7 minutes. Analytes were quantitated using the total ion current of a single reaction monitoring experiment as compared to the response of FB1, FB2, and FB3 standards (provided by Ronald Plattner, National Center for Agricultural Utilization Research, USDA-ARS, Peoria, IL; > 95%). The experiment was conducted a minimum of three times.

### GYAM plate assay

*F*. *verticillioides* strains were inoculated and grown for 7 days at 27°C on 20 mL GYAM 1.5% agar plates [41]. A peri-marginal 6-mm diameter agar plug of each strain was transferred to the center of fresh 20 mL GYAM plates and allowed to grow in the dark at 27°C. Growth phenotypes were visually inspected daily until 9 days post-inoculation. Three biological replicates were prepared for each strain.

### Public availability of data

RNA-Seq data were deposited in NCBI’s Gene Expression Omnibus (GEO) and are accessible through GEO Series accession GSE116351 (http://www.ncbi.nlm.nih.gov/geo/). The reference genome *Fusarium verticillioides* 7600 (ASM14955V1) was obtained from NCBI genome database (https://www.ncbi.nlm.nih.gov/genome/) [13].

## Supporting information

S1 Fig Deletion of genes in this study was confirmed by PCR. To confirm the deletion of target genes, four PCR reactions were performed for each mutant to determine 1) the presence of hygromycin resistance cassette (HRC); 2) the absence of the open reading frame (ORF); 3) the homologous recombination at the 5’ flank; 4) the homologous recombination at the 3’ flank. L: 1kb ladder (New England BioLabs Inc.); O: target gene ORF; H: HRC; 5’: 5’ flank; 3’: 3’ flank. Verification was performed for (**A**) Δ*FvABC3* (FVEG_11089) mutants, (**B**) Δ*FvZBD1* (FVEG_00314) mutants, and (**C**) the Δ*FvZEAR* (FVEG_11090) mutant; M-3125 genomic DNA, molecular grade water, and ectopic transformed strains served as the control DNA templates.
(TIFF)

S2 Fig Deletion mutants possessed a single genomic copy of the hygromycin resistance cassette. The copy number of the hygromycin resistance cassette (HRC) in the mutants was determined by qPCR of extracted genomic DNA. M-3125 and ΔFVEG_08294 served as null and single-copy controls, respectively. ΔFVEG_08294 is a deletion mutant with only a single HRC as previously determined using Southern hybridization. The data were normalized to the reference β-tubulin gene (FVEG_04081) and calculated via the 2^-ΔΔCt^ method [40]. The ΔCt standard error is indicated by error bar. Copy number determination was performed for (A) Δ*FvABC3* and Δ*FvZEAR*, and (B) Δ*FvZBD1* mutants. Three technical replicates were prepared for each strain. There were no significant differences in abundance levels for the HRC among Δ*FvABC3*, Δ*FvZEAR*, Δ*FvZBD1*, and the ΔFVEG_08294 single-copy control (two-tailed Mann Whitney Wilcoxon test, p-value < 0.05).
(TIFF)

S3 Fig Deletion of *FvABC3* in *F*. *verticillioides* did not alter fungal virulence on maize seedlings. Fifty Silver Queen maize seeds were inoculated with 10^4^/mL conidial suspensions for each of the five different *F*. *verticillioides* strains prior to planting. An uninoculated control treated with sterile water was also included. Plants were grown for 14 days before measuring their heights and counting germinated seeds. The experiment was repeated three times with three technical replicates each. Trials consistently showed no differences in virulence between M-3125 and the *FvABC3* deletion mutants. Data from one trial was plotted for representation. (**A**) Histogram showing the mean height of seedlings. Numbers on X-axis correspond to the following treatments: **1**, sterile water control; **2**, M-3125; **3**, Δ*FvABC3*-1; **4**, Δ*FvABC3*-2; **5**, Δ*FvABC3*-1::C-1; **6**, Δ*FvABC3*-1::C-1. Statistical analysis was conducted with the two-tailed Mann Whitney Wilcoxon test. (**B**) Phenotypic representation of seeding growth among treatments. Numbers below the pots correspond to those in (A). (**C**) Two-dimensional visualization of seedling growth among different treatments. Each dot represents a technical replicate of a particular treatment. Total height (cm) of all seedlings per pot is denoted on the Y-axis, and the X-axis shows the number of germinated seeds per pot.
(TIFF)

S4 Fig Deletion of *FvABC3* did not impact fumonisin production in GYAM liquid cultures. Two milliliters of GYAM liquid medium in snap-cap tubes with loose caps were inoculated with 10^4^ spores of each strain and cultured in dark at 250 rpm, 27°C for 7 days. Fumonisin concentrations were determined by LC-MS and normalized to the vacuum-desiccated fungal mass weight, as indicated on the Y-axis. Statistical analyses performed with two-tailed Mann Whitney Wilcoxon test showed no significant differences (p-value < 0.05), in terms of fumonisin production, between deletion mutants and M-3125. FB1/FB2/FB3 represent fumonisin B1/B2/B3.
(TIFF)

S5 Fig Deletion of *FvZBD1* resulted in increased sensitivity to pyrrocidine B. Strains were monitored for 100 hours in PDB media amended with pyrrocidine B at 10 μg/mL. OD_600_ measurements taken every 2 hours were plotted (mean ± standard deviation). FRC M-3125 serves as the control (black curve). Two *FvZBD1* deletion mutants are shown in orange, and two complemented strains in green.
(TIFF)

S6 Fig FvABC3 shows typical ABC transporter transmembrane domain arrangement. (A) Eleven transmembrane domains were predicted with the TMHMM web server (http://www.cbs.dtu.dk/services/TMHMM/). The X-axis refers to the position of amino acid sequences, and the Y-axis corresponds to the probability of being a transmembrane domain. (B) The predicted transmembrane topology of FvABC3 amino acid sequence by the CAMPS (Computational Analysis of the Membrane Protein Space) database (http://webclu.bio.wzw.tum.de:18080/CAMPS2.0/index.jsp) [43]. The polarity of amino acids is marked by the color index.
(TIFF)

S1 Table Summary of RNA-Seq mapping results. (DOCX)

S2 Table Additional primers in this study. (DOCX)

S3 Table qRT-PCR confirmation of differential expression of genes targeted for functional characterization. (DOCX)
