# Supplementary figures and images for: Pyrrocidine, a molecular off switch for fumonisin biosynthesis
Source: PLoS Pathog. 2020 Jul 6;16(7):e1008595. doi: 10.1371/journal.ppat.1008595 (PMC7377494; doi:10.1371/journal.ppat.1008595)

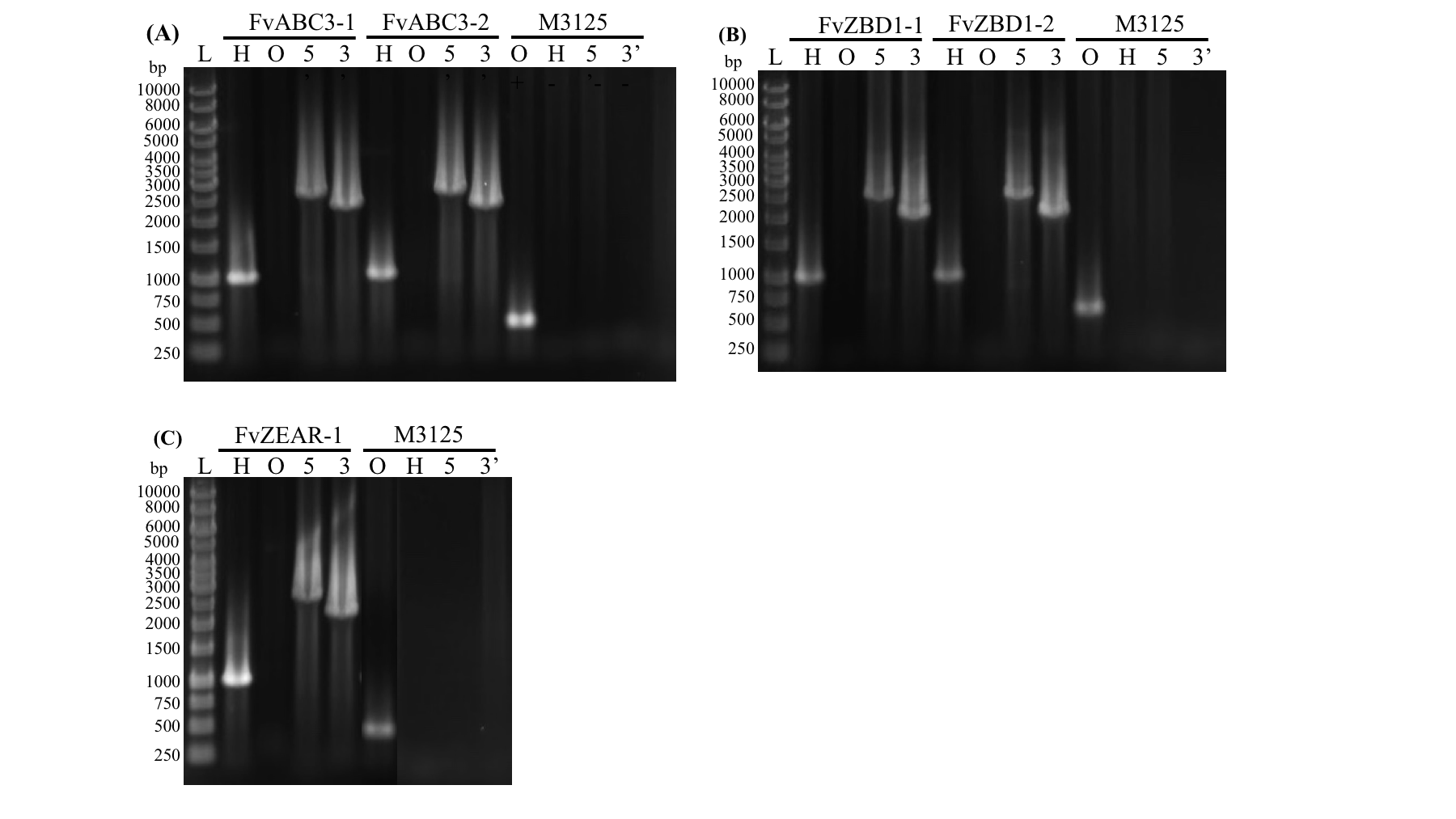

Supplement: S1 Fig — To confirm the deletion of target genes, four PCR reactions were performed for each mutant to determine 1) the presence of hygromycin resistance cassette (HRC); 2) the absence of the open reading frame (ORF); 3) the homologous recombination at the 5’ flank; 4) the homologous recombination at the 3’ flank. L: 1kb ladder (New England BioLabs Inc.); O: target gene ORF; H: HRC; 5’: 5’ flank; 3’: 3’ flank. Verification was performed for (A) ΔFvABC3 (FVEG_11089) mutants, (B) ΔFvZBD1 (FVEG_00314) mutants, and (C) the ΔFvZEAR (FVEG_11090) mutant; M-3125 genomic DNA, molecular grade water, and ectopic transformed strains served as the control DNA templates. (TIFF) [file ppat.1008595.s001.tiff]

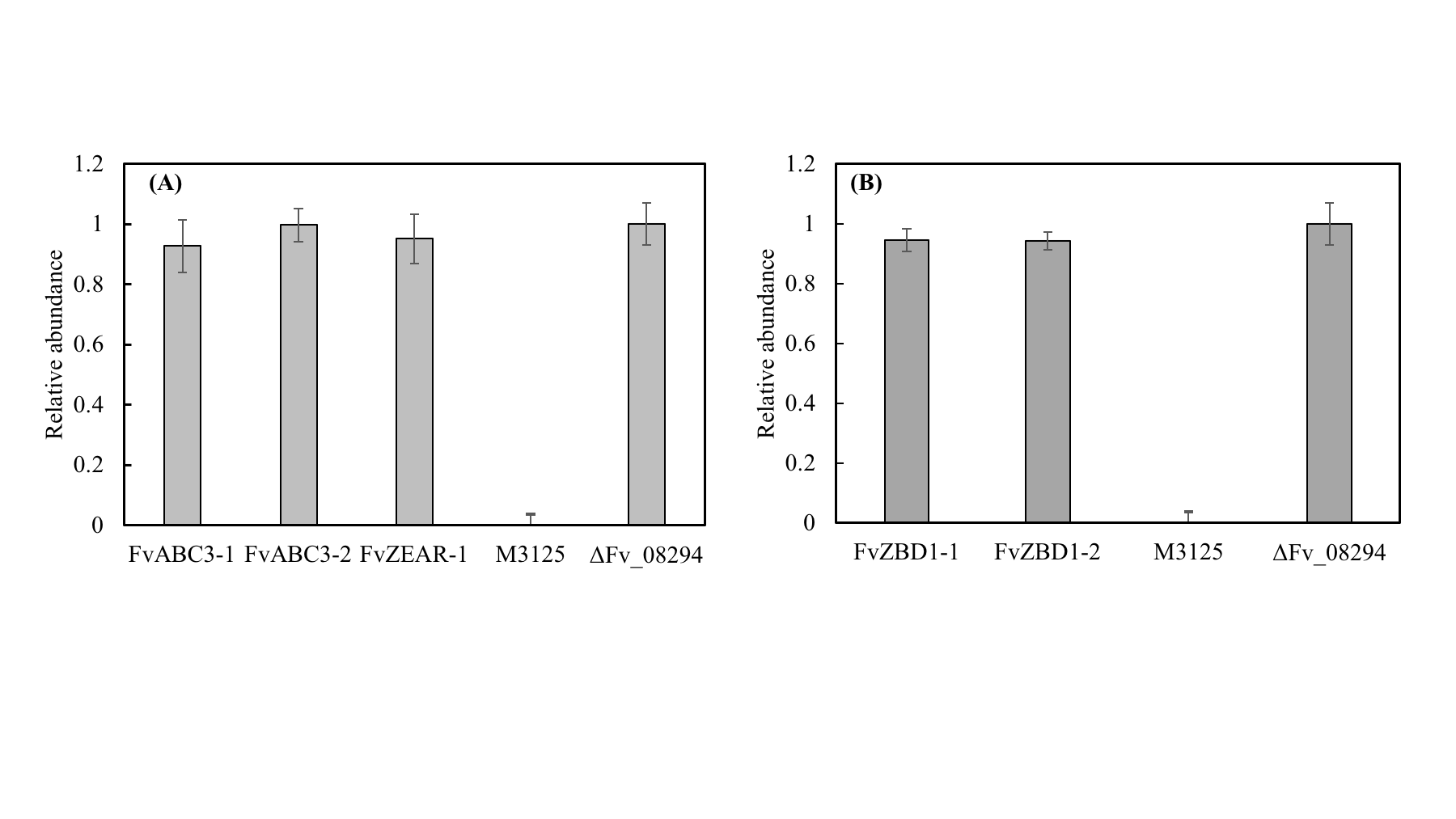

Supplement: S2 Fig — The copy number of the hygromycin resistance cassette (HRC) in the mutants was determined by qPCR of extracted genomic DNA. M-3125 and ΔFVEG_08294 served as null and single-copy controls, respectively. ΔFVEG_08294 is a deletion mutant with only a single HRC as previously determined using Southern hybridization. The data were normalized to the reference β-tubulin gene (FVEG_04081) and calculated via the 2-ΔΔCt method [40]. The ΔCt standard error is indicated by error bar. Copy number determination was performed for (A) ΔFvABC3 and ΔFvZEAR, and (B) ΔFvZBD1 mutants. Three technical replicates were prepared for each strain. There were no significant differences in abundance levels for the HRC among ΔFvABC3, ΔFvZEAR, ΔFvZBD1, and the ΔFVEG_08294 single-copy control (two-tailed Mann Whitney Wilcoxon test, p-value < 0.05). (TIFF) [file ppat.1008595.s002.tiff]

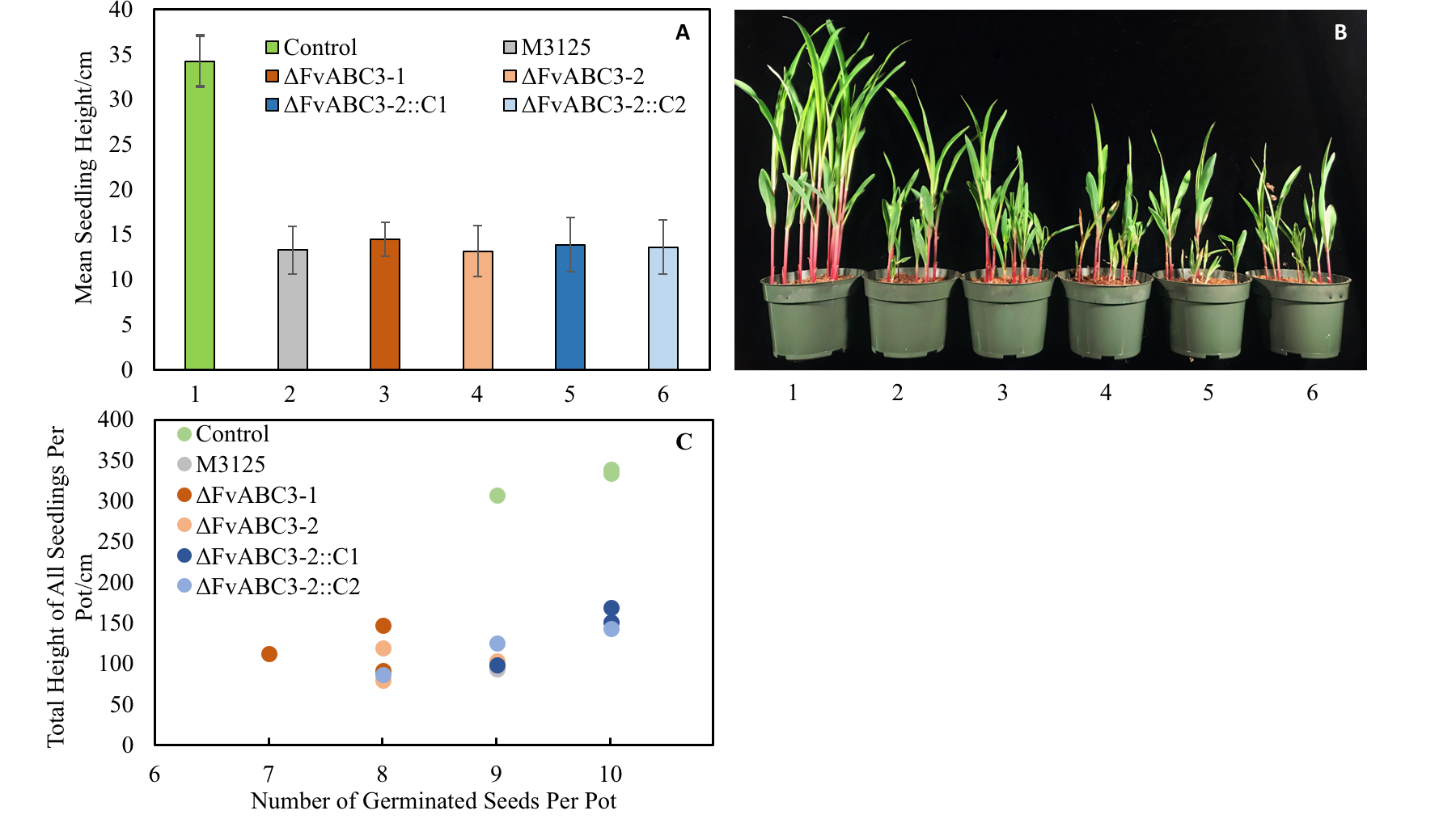

Supplement: S3 Fig — Fifty Silver Queen maize seeds were inoculated with 104/mL conidial suspensions for each of the five different F. verticillioides strains prior to planting. An uninoculated control treated with sterile water was also included. Plants were grown for 14 days before measuring their heights and counting germinated seeds. The experiment was repeated three times with three technical replicates each. Trials consistently showed no differences in virulence between M-3125 and the FvABC3 deletion mutants. Data from one trial was plotted for representation. (A) Histogram showing the mean height of seedlings. Numbers on X-axis correspond to the following treatments: 1, sterile water control; 2, M-3125; 3, ΔFvABC3-1; 4, ΔFvABC3-2; 5, ΔFvABC3-1::C-1; 6, ΔFvABC3-1::C-1. Statistical analysis was conducted with the two-tailed Mann Whitney Wilcoxon test. (B) Phenotypic representation of seeding growth among treatments. Numbers below the pots correspond to those in (A). (C) Two-dimensional visualization of seedling growth among different treatments. Each dot represents a technical replicate of a particular treatment. Total height (cm) of all seedlings per pot is denoted on the Y-axis, and the X-axis shows the number of germinated seeds per pot. (TIFF) [file ppat.1008595.s003.tiff]

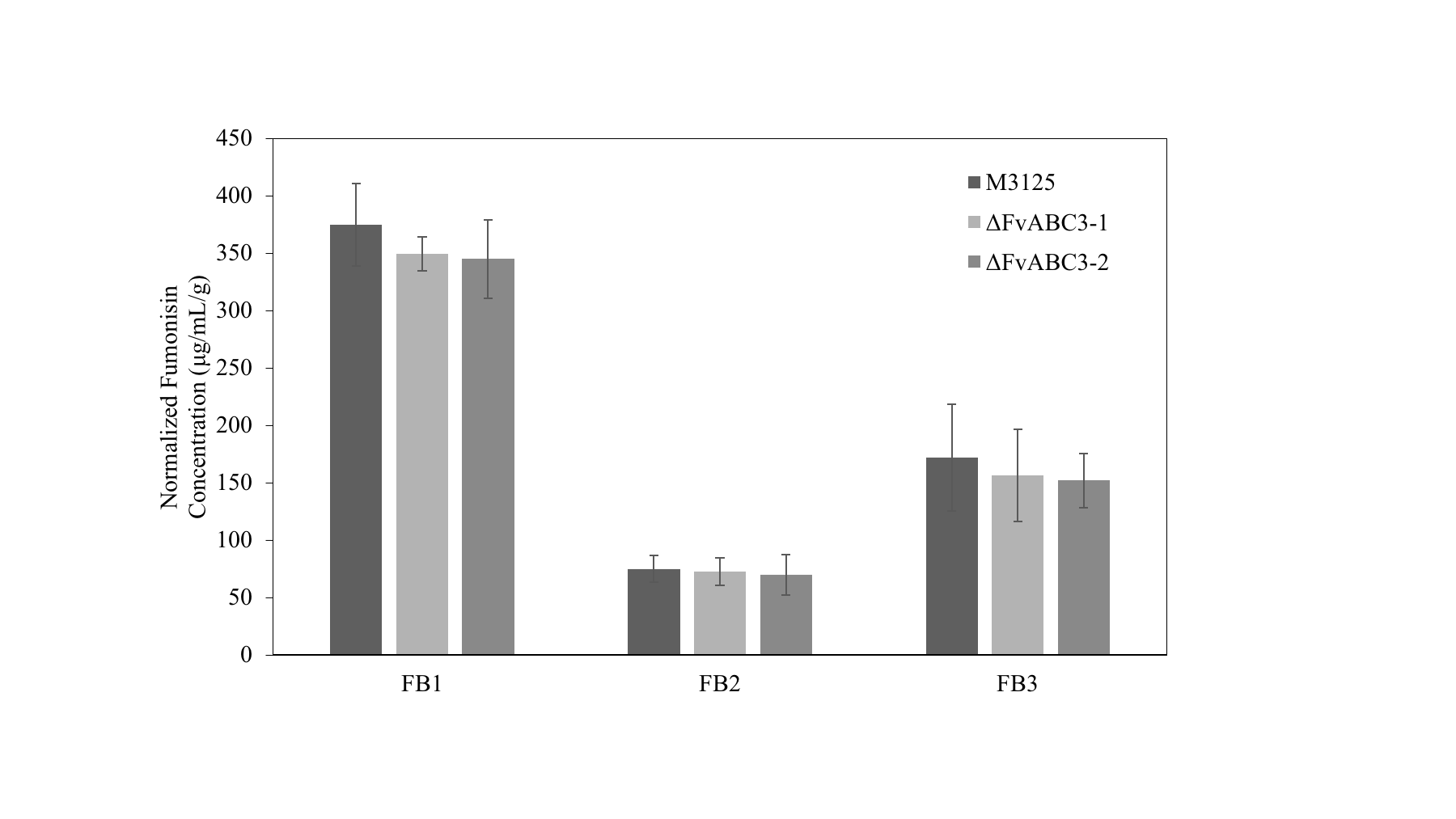

Supplement: S4 Fig — Two milliliters of GYAM liquid medium in snap-cap tubes with loose caps were inoculated with 104 spores of each strain and cultured in dark at 250 rpm, 27°C for 7 days. Fumonisin concentrations were determined by LC-MS and normalized to the vacuum-desiccated fungal mass weight, as indicated on the Y-axis. Statistical analyses performed with two-tailed Mann Whitney Wilcoxon test showed no significant differences (p-value < 0.05), in terms of fumonisin production, between deletion mutants and M-3125. FB1/FB2/FB3 represent fumonisin B1/B2/B3. (TIFF) [file ppat.1008595.s004.tiff]

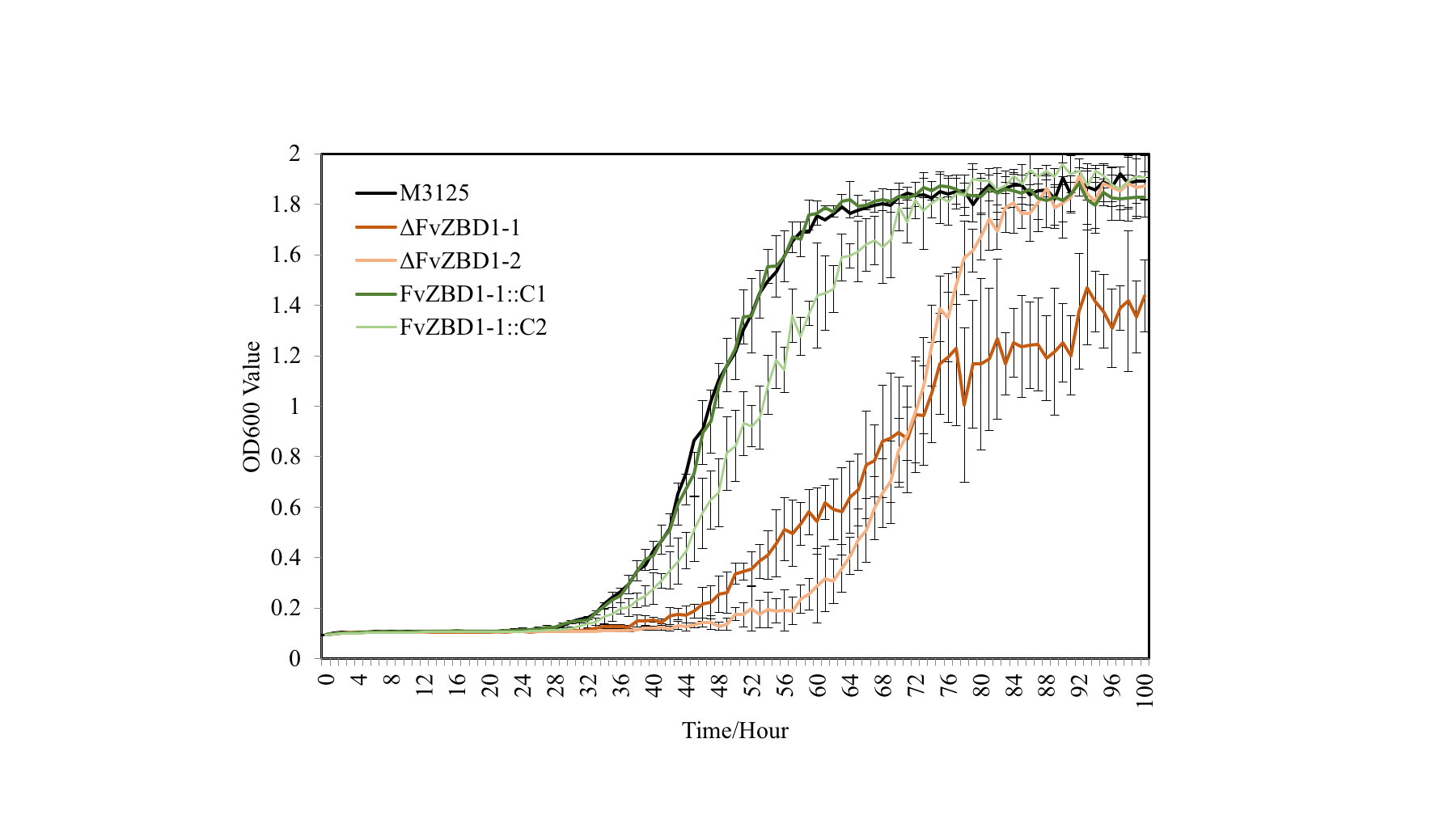

Supplement: S5 Fig — Strains were monitored for 100 hours in PDB media amended with pyrrocidine B at 10 μg/mL. OD600 measurements taken every 2 hours were plotted (mean ± standard deviation). FRC M-3125 serves as the control (black curve). Two FvZBD1 deletion mutants are shown in orange, and two complemented strains in green. (TIFF) [file ppat.1008595.s005.tiff]

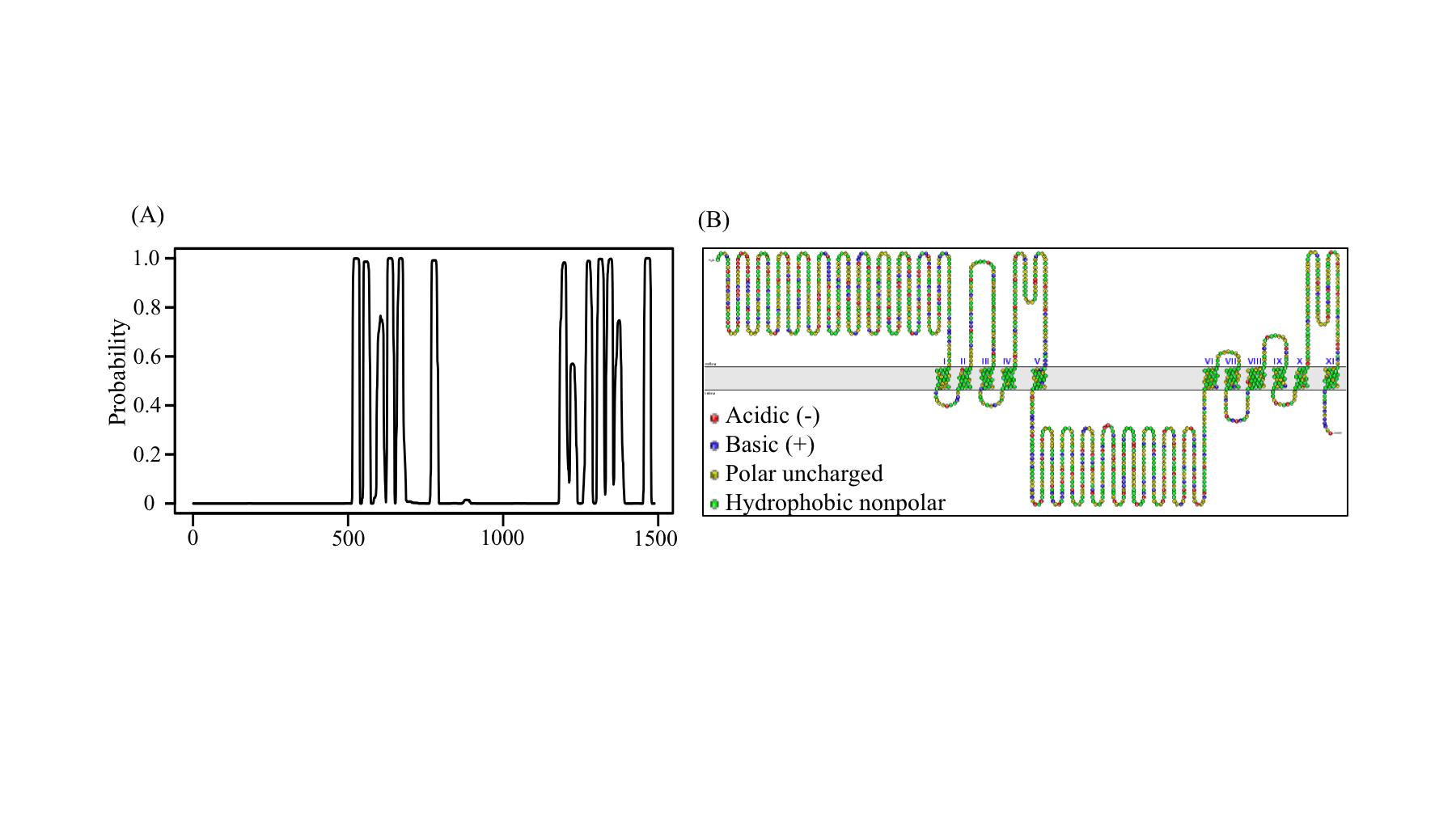

Supplement: S6 Fig — (A) Eleven transmembrane domains were predicted with the TMHMM web server (http://www.cbs.dtu.dk/services/TMHMM/). The X-axis refers to the position of amino acid sequences, and the Y-axis corresponds to the probability of being a transmembrane domain. (B) The predicted transmembrane topology of FvABC3 amino acid sequence by the CAMPS (Computational Analysis of the Membrane Protein Space) database (http://webclu.bio.wzw.tum.de:18080/CAMPS2.0/index.jsp) [43]. The polarity of amino acids is marked by the color index. (TIFF) [file ppat.1008595.s006.tiff]
