# Supplementary material for: Pyrrocidine, a molecular off switch for fumonisin biosynthesis
Source: PLoS Pathog. 2020 Jul 6;16(7):e1008595. doi: 10.1371/journal.ppat.1008595 (PMC7377494; doi:10.1371/journal.ppat.1008595)
Supplement: S1 Table — (DOCX) [file ppat.1008595.s007.docx]

**Supplemental Table 1** Summary of RNA-Seq mapping results

| **Sample**  **treatment*** | **Number of**  **raw reads** | **Number of**  **mapped reads** | **Mapping**  **rates** |
| --- | --- | --- | --- |
| Control-1 | 16342483 | 15943588 | 97.6% |
| Control-2 | 14185251 | 13921851 | 98.1% |
| Control-3 | 19119430 | 18873749 | 98.7% |
| PA5-1 | 17239023 | 17042978 | 98.9% |
| PA5-2 | 17788944 | 17377585 | 97.7% |
| PA5-3 | 15630242 | 15186301 | 97.2% |
| PB20-1 | 15083542 | 14906800 | 98.8% |
| PB20-2 | 21877419 | 21492342 | 98.2% |
| PB20-3 | 21183781 | 20818211 | 98.3% |

* Three biological replicates were prepared for 3 treatments including 1) DMSO control, 2) PA5, pyrrocidine A at 5 μg/mL, and 3) PB20, pyrrocidine B at 20 μg/mL.
