# Supplementary material for: Pyrrocidine, a molecular off switch for fumonisin biosynthesis
Source: PLoS Pathog. 2020 Jul 6;16(7):e1008595. doi: 10.1371/journal.ppat.1008595 (PMC7377494; doi:10.1371/journal.ppat.1008595)
Supplement: S2 Table — (DOCX) [file ppat.1008595.s008.docx]

**Supplemental Table 2** Additional primers in this study

| **Index** | **Primer Name** | **Primer Sequence** |
| --- | --- | --- |
| HP1/29 | FVEG_01675_OSC1 | GGGGACAGCTTTCTTGTACAAAGTGGAACCGACGAATTAATGACTCTATCT |
| HP1/30 | FVEG_01675_OSC2 | GGGGACTGCTTTTTTGTACAAACTTGTGAACCAGTTATGGTAGGAGTTAAT |
| HP1/31 | FVEG_01675_OSC3 | GGGGACAACTTTGTATAGAAAAGTTGTTCATGGATATTACAATAAGCCAAGAG |
| HP1/32 | FVEG_01675_OSC4 | GGGGACAACTTTGTATAATAAAGTTGTCAATTAATCGAGCATCAACTCAC |
| HP1/33 | FVEG_01675_Orf_F | GGCATATTTGGGATGAAAGTAGTA |
| HP1/34 | FVEG_01675_Orf_R | CTTTCAAAGCTGTTCGGAATTATC |
| HP1/35 | FVEG_07235_OSC1 | GGGGACAGCTTTCTTGTACAAAGTGGAAGCAGGATTTGAGTTGTAGTATTTAG |
| HP1/36 | FVEG_07235_OSC2 | GGGGACTGCTTTTTTGTACAAACTTGTGAGTTGAGCATAAATGAGGAAAC |
| HP1/37 | FVEG_07235_OSC3 | GGGGACAACTTTGTATAGAAAAGTTGTTCGTTGTAGTAAATTCCAAGCTCTA |
| HP1/38 | FVEG_07235_OSC4 | GGGGACAACTTTGTATAATAAAGTTGTTGATTTCCTCAAACATGTCTCTC |
| HP1/39 | FVEG_07235_Orf_F | CCTGAGTATTACATGGCTTCTAC |
| HP1/40 | FVEG_07235_Orf_R | CTACCTCAAAGTAGTCGAATGAG |
| HP1/41 | FVEG_09038_OSC1 | GGGGACAGCTTTCTTGTACAAAGTGGAACCCGATCTATCAGTGGAGTATTA |
| HP1/42 | FVEG_09038_OSC2 | GGGGACTGCTTTTTTGTACAAACTTGTAGAGAATATTGCTGAACTCTTGG |
| HP1/43 | FVEG_09038_OSC3 | GGGGACAACTTTGTATAGAAAAGTTGTTACTCATCCTAATGAATAGAGAACG |
| HP1/44 | FVEG_09038_OSC4 | GGGGACAACTTTGTATAATAAAGTTGTTTCACTTAGCATCTCAACAACTC |
| HP1/45 | FVEG_09038_Orf_F | CGTGTAACTCAGGACTTCATTAT |
| HP1/46 | FVEG_09038_Orf_R | TTAGTCTCTATTTGACCCTGAAAG |
| HP1/47 | FVEG_13271_OSC1 | GGGGACAGCTTTCTTGTACAAAGTGGAAGGATATACAGTCAAGGTCGAATAC |
| HP1/48 | FVEG_13271_OSC2 | GGGGACTGCTTTTTTGTACAAACTTGTGATGTCAGGTAGTACAAAGGAAG |
| HP1/49 | FVEG_13271_OSC3 | GGGGACAACTTTGTATAGAAAAGTTGTTGTGTAGGGTGATGATTGGTAATA |
| HP1/50 | FVEG_13271_OSC4 | GGGGACAACTTTGTATAATAAAGTTGTTGTCTCCTTTCTCTTGTCTTATAG |
| HP1/51 | FVEG_13271_Orf_F | CTCCTTTCAGTGCGAGTAATAG |
| HP1/52 | FVEG_13271_Orf_R | AGCCGTCTTAGCAGATATATAAAG |
| HP1/53 | FVEG_13322_OSC1 | GGGGACAGCTTTCTTGTACAAAGTGGAAACTTACAGAAGTATGTATCTGAGC |
| HP1/54 | FVEG_13322_OSC2 | GGGGACTGCTTTTTTGTACAAACTTGTGAGGCTTCGTCACTTAACTATAA |
| HP1/55 | FVEG_13322_OSC3 | GGGGACAACTTTGTATAGAAAAGTTGTTCATTCAGTTGGCAGATTCATTTAC |
| HP1/56 | FVEG_13322_OSC4 | GGGGACAACTTTGTATAATAAAGTTGTGAAGTTATCTGGAAGAGCCTTAAT |
| HP1/57 | FVEG_13322_Orf_F | GTATATAAAGGCTAATCCGCAATG |
| HP1/58 | FVEG_13322_Orf_R | GATGATGACGTCTAGCTTAGTAAT |
| HP1/59 | FVEG_17422_OSC1 | GGGGACAGCTTTCTTGTACAAAGTGGAACCTGTTAACACTTCCTTGTTAGA |
| HP1/60 | FVEG_17422_OSC2 | GGGGACTGCTTTTTTGTACAAACTTGTCAGAAACCTCATAAACTTCAACTC |
| HP1/61 | FVEG_17422_OSC3 | GGGGACAACTTTGTATAGAAAAGTTGTTATATTCCGTCGCTCTAATAACTAC |
| HP1/62 | FVEG_17422_OSC4 | GGGGACAACTTTGTATAATAAAGTTGTCATCATAGTCCTGTTACTCAATCT |
| HP1/63 | FVEG_17422_Orf_F | CTGCATGGGATAACAGGTAAG |
| HP1/64 | FVEG_17422_Orf_R | CTCTCTCAAAGGCGCAATAG |
| HP1/65 | FVEG_17625_OSC1 | GGGGACAGCTTTCTTGTACAAAGTGGAAGTACTTGAAGATTATCGACCTAGAG |
| HP1/66 | FVEG_17625_OSC2 | GGGGACTGCTTTTTTGTACAAACTTGTGTGGAGTCCGAATATAACTGATTA |
| HP1/67 | FVEG_17625_OSC3 | GGGGACAACTTTGTATAGAAAAGTTGTTGACGATGGTGGGTAAATATCTATG |
| HP1/68 | FVEG_17625_OSC4 | GGGGACAACTTTGTATAATAAAGTTGTCTAGATTGAGACATGAACGTAACA |
| HP1/69 | FVEG_17625_Orf_F | CAAGCGCTCTACCTAGTTTC |
| HP1/70 | FVEG_17625_Orf_R | GCCTCATTCGTGTACTTTACT |
