# Supplementary material for: Pyrrocidine, a molecular off switch for fumonisin biosynthesis
Source: PLoS Pathog. 2020 Jul 6;16(7):e1008595. doi: 10.1371/journal.ppat.1008595 (PMC7377494; doi:10.1371/journal.ppat.1008595)
Supplement: S3 Table — (DOCX) [file ppat.1008595.s009.docx]

**Supplemental Table 3** qRT-PCR confirmation of differential expression of genes targeted for functional characterization.

| Gene | Normalized Expression of qRT-PCR by 2^(ΔΔCT) method | Normalized Std. Error |
| --- | --- | --- |
| FVEG_00314 | 181.940 | 22.920 |
| FVEG_01675 | 50.757 | 5.982 |
| FVEG_07325 | 41.002 | 5.178 |
| FVEG_09038 | 77.699 | 8.286 |
| FVEG_11089 | 705.051 | 84.510 |
| FVEG_11090 | 17.727 | 1.963 |
| FVEG_13271 | 20.266 | 2.108 |
| FVEG_13322 | 496.470 | 67.672 |
| FVEG_17422 | 246.050 | 31.745 |
| FVEG_17625 | 4.110 | 0.664 |
